# Double vision: 2D and 3D mosquito trajectories can be as valuable for behaviour analysis via machine learning

**DOI:** 10.1186/s13071-024-06356-9

**Published:** 2024-07-01

**Authors:** Yasser Mehmood Qureshi, Vitaly Voloshin, Catherine Elizabeth Towers, James Anthony Covington, David Peter Towers

**Affiliations:** 1 School of Engineering, University of Warwick, Coventry, CV4 7AL UK; 2 School of Biological and Behavioural Sciences, Queen Mary University of London, London, E1 4NS UK

**Keywords:** Mosquito tracking, Imaging systems, Trajectories, Machine learning, Behaviour

## Abstract

**Background:**

Mosquitoes are carriers of tropical diseases, thus demanding a comprehensive understanding of their behaviour to devise effective disease control strategies. In this article we show that machine learning can provide a performance assessment of 2D and 3D machine vision techniques and thereby guide entomologists towards appropriate experimental approaches for behaviour assessment. Behaviours are best characterised via tracking—giving a full time series of information. However, tracking systems vary in complexity. Single-camera imaging yields two-component position data which generally are a function of all three orthogonal components due to perspective; however, a telecentric imaging setup gives constant magnification with respect to depth and thereby measures two orthogonal position components. Multi-camera or holographic techniques quantify all three components.

**Methods:**

In this study a 3D mosquito mating swarm dataset was used to generate equivalent 2D data via telecentric imaging and a single camera at various imaging distances. The performance of the tracking systems was assessed through an established machine learning classifier that differentiates male and non-male mosquito tracks. SHAPs analysis has been used to explore the trajectory feature values for each model.

**Results:**

The results reveal that both telecentric and single-camera models, when placed at large distances from the flying mosquitoes, can produce equivalent accuracy from a classifier as well as preserve characteristic features without resorting to more complex 3D tracking techniques.

**Conclusions:**

Caution should be exercised when employing a single camera at short distances as classifier balanced accuracy is reduced compared to that from 3D or telecentric imaging; the trajectory features also deviate compared to those from the other datasets. It is postulated that measurement of two orthogonal motion components is necessary to optimise the accuracy of machine learning classifiers based on trajectory data. The study increases the evidence base for using machine learning to determine behaviours from insect trajectory data.

**Graphical Abstract:**

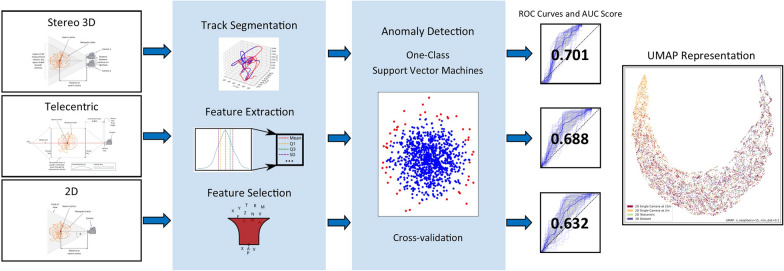

**Supplementary Information:**

The online version contains supplementary material available at 10.1186/s13071-024-06356-9.

## Background

Mosquito-borne diseases present a significant risk to human health, with nearly 700 million cases and 750,000 deaths reported globally each year [1]. To combat these diseases, it is crucial to understand the behaviour of mosquitoes. Tracking mosquitoes produces trajectories that can return valuable insights into their flight behaviour and has already led to significant advances in disease prevention. For instance, early studies on mosquito trajectories led to the development of an improved insecticide-treated net (ITN) design that provides better protection against disease transmission [2]. Further research on mosquito behaviour is likely to lead to other such improvements.

Previously, many tracking studies involved manual processing to capture behaviours, with a number of examples concerning mosquitoes [3–5]. However, advancements in high-resolution cameras, computational power and computer vision technology have enabled automated tracking of behaviour [6]. Typically, this involves using cameras to capture videos or images that are subsequently processed to identify the mosquitoes or objects of interest. To facilitate accurate tracking, experiments rely on a clear contrast between the insect and background, achieved by illumination control. Appropriate lighting can be achieved using front, back or side illumination with artificial sources where the wavelength is normally selected in an insect blind region of the spectrum; in some cases natural lighting from the sun can be used effectively [7, 8]. Tracking individual insects entails analysing the contrast differences within the images. By applying appropriate thresholds, the objects of interest are accurately segmented from the background.

**Fig. 1:**
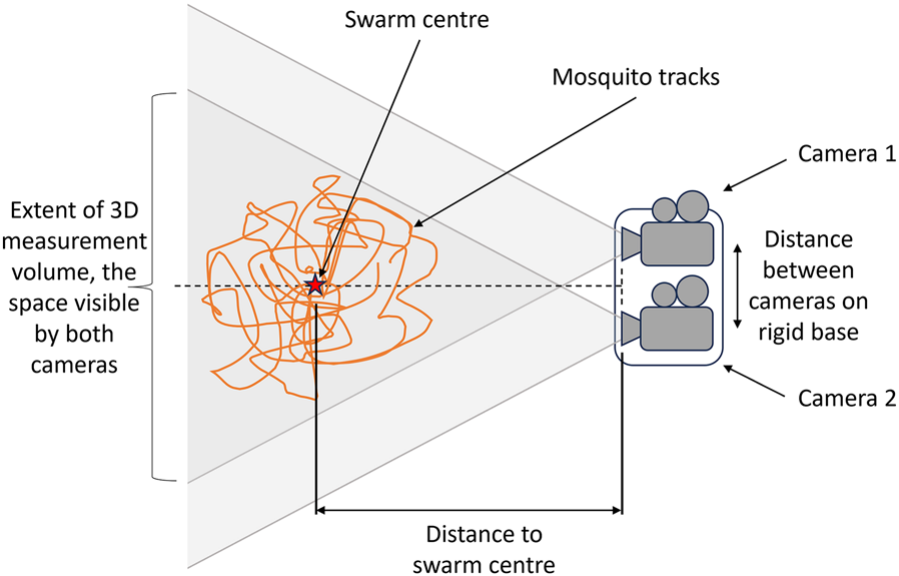
Schematic of stereo camera setup for 3D mosquito tracking illustrating the boundaries of the space imaged by both cameras and where 3D measurements are possible

**Fig. 2:**
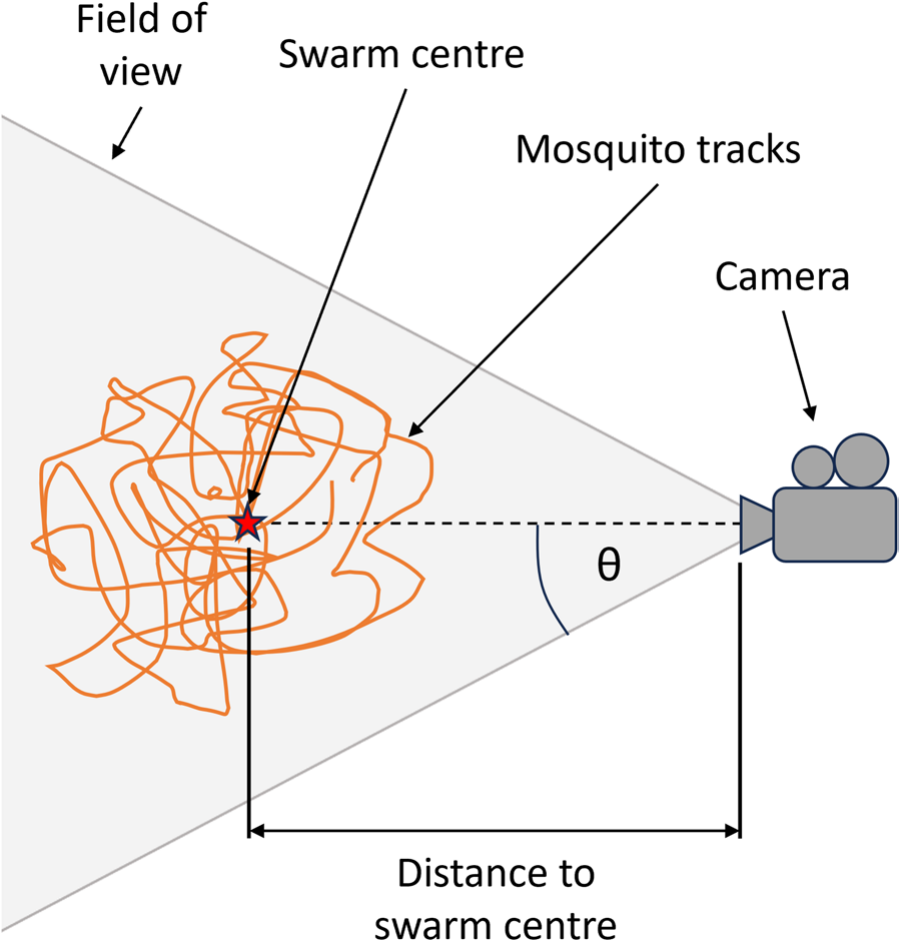
Schematic of single-camera setup for 2D mosquito tracking. The imaged volume is determined by the angular field of view, θ, and hence increases with distance from the camera

Insect behaviour can be quantified using two-dimensional (2D) or three-dimensional (3D) tracking systems. Three-dimensional tracking provides full quantitative measurement of the three orthogonal components of an object’s position and movement in 3D space. This is at the expense of a more complex imaging setup and hence higher cost. The most widely used approach for 3D tracking is stereo vision with a pair of rigidly coupled cameras (Fig. 1) [7]. The camera separation is one of the main factors that determines the resolution of the depth information with respect to the cameras, increased separation giving improved resolution at the expense of less correspondence between the camera views, a larger setup and needing a more rigid mechanical coupling between the cameras. Camera calibration is crucial, particularly when attempting to construct 3D trajectories from stereo cameras. This process involves establishing a relationship between the 2D coordinates obtained from each camera and the 3D coordinates of markers in a known pattern from a set of calibration frames. Typically, stereo camera calibration has to be performed in situ and also compensates for lens distortion [6]. In contrast, 2D tracking recovers the motion of a body from the projection of its position onto the 2D image plane of a single camera and some information is lost (Fig. 2). The two-component information obtained, in general, is a combination of the three orthogonal position components due to perspective projection. The field of view has an angular limit, determined by the camera lens. Hence, a specific mosquito movement at the front and back of the measurement volume will give differing results in pixels on the camera. Fortunately, several software packages are available that facilitate automated tracking. These packages provide functionalities for image pre-processing, object identification, and trajectory analysis, streamlining the tracking process and reducing the manual effort required [9–11].

**Fig. 3:**
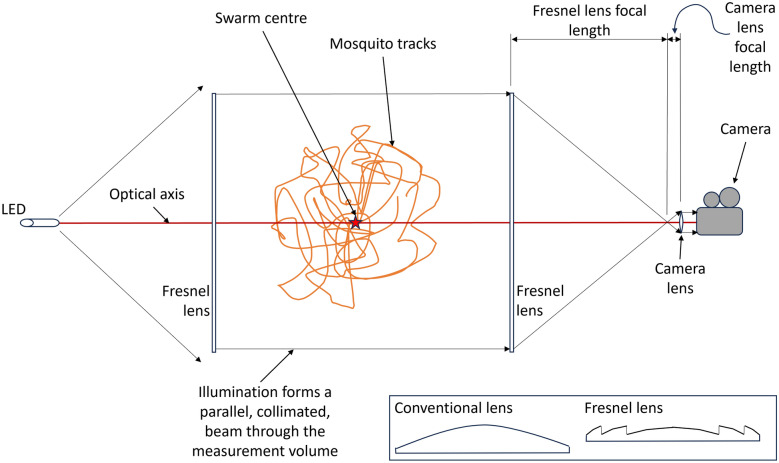
Schematic of a single-camera telecentric setup for measuring two orthogonal components of mosquito movement. The mosquitoes are back-lit from the LED on the left hand side and are observed as shadows on the camera

Telecentric imaging was introduced for single-camera, 2D measurement applications as an object appears at the same size irrespective of its position along the optical axis (Fig. 3) [12]. It employs a lens with aperture matching the field of view, and Fresnel lenses enable large, metre-scale applications (see inset in Fig. 3). The telecentric arrangement is achieved by spacing the two lenses on the camera side by a distance equal to the sum of their focal lengths. This geometry removes the perception of depth and eliminates perspective distortion [12, 13]. Wide-angle LED sources with a large aperture Fresnel lens for illumination makes telecentric imaging well suited for indoor recordings.

In recent studies, researchers have explored 2D and 3D trajectories, shedding light on their respective merits and limitations. A notable investigation focused on zebrafish behaviour, where a comparison was made between 3D and 2D tracking [14]. To capture the zebrafish movements, two cameras were positioned to view orthogonal planes within a large water tank. Videos were processed into frames and analysed with a 3D multi-target tracking algorithm [15] resulting in the quantification of a range of essential behavioural characteristics. Intriguingly, the analysis revealed consistent underestimation of these behavioural features when relying solely on 2D views. This discrepancy can be attributed to the lack of the extra dimension provided by 3D tracking, which offers a more comprehensive understanding of the zebrafish's rich behavioural repertoire. Consequently, it was concluded that collecting and analysing 3D trajectories was a necessary overhead, despite the use of multiple cameras and an increased computational load. Furthermore, an additional finding emerged, indicating that a 3D approach requires fewer subjects compared to a 2D approach to obtain comparable statistical results. More recently, stereo-based 3D tracking has been instrumental in understanding moth behaviour in attraction to artificial light revealing that dorsal tilting is responsible for the seemingly erratic flight of the moth around a light source [16].

Tracking techniques have greatly advanced our understanding of mosquito behaviours. Butail et al. [8] (2012) used a stereo camera system to construct and validate 3D trajectories of wild *Anopheles gambiae*. This research revealed insights into male mosquito motion [17]. Building upon these findings, a more recent study [18] focused on classifying the disparities in mosquito behaviour between male and non-male (females and mating couples). By utilising explainable artificial intelligence (XAI), the study explored the dissimilarities among these classes, reinforcing existing knowledge about the behaviour of male mosquitoes within mating swarms. XAI showed that females and mating couples (non-males) tend to exhibit extreme, high and low, values for velocity and acceleration features (kinematic characteristics) perhaps reflecting the increased energy availability in females through blood feeding and the more chaotic movement of mating couples. The paper shows the utility of machine learning, and XAI techniques in particular, to extract behaviour insights from 3D trajectory information. Parker et al. [19], examined mosquito behaviours around human baited bednets in the field using 2D imaging of *Anopheles gambiae* mosquitoes. Here, a pair of identical recording systems were used ‘side by side’ to expand the field of view and telecentric imaging was utilised (as shown in Fig. 3) to produce an accurate projection of two orthogonal components of motion onto the image plane. This research identified four distinct behavioural modes—swooping, visiting, bouncing, and resting—using bespoke algorithms based on entomologist expertise. Furthermore, it was observed that mosquitoes possess the ability to detect nets, including unbaited untreated ones. These findings contributed to the understanding of mosquito interaction with ITNs.

Tracking in combination with AI techniques has also been used to examine behaviours of other insects. Machraoui et al. [20] used 2D imaging, tracking and feature extraction with supervised learning models to differentiate sandflies from other insects with accuracies of circa 88% for support vector machine and artificial neural network models on an optimised feature set.

In this article, we explore the relative merits of 2D and 3D mosquito tracking when classifying and interpreting behaviours via machine learning. We present a comparative analysis among 3D trajectories, 2D telecentric (removing one orthogonal component) and 2D single-camera data with perspective distortion, all derived from the same dataset, to assess the advantages and limitations of these tracking approaches. Analogous features are determined for each of these datasets, and the accuracy of the machine learning classifier provides a useful quantitative metric to assess the outcomes and XAI enables interpretation of behaviours. We hypothesise that 3D tracking and 2D telecentric tracking will return similar results, despite the loss of the additional information in the third dimension. We further hypothesise that a single-camera tracking system will return lower performance due to perspective effects and lens distortion. A deeper understanding of the strengths and weaknesses of 2D and 3D mosquito tracking will enable researchers to make informed decisions regarding experiment design. Overall, our research endeavours to advance the field of mosquito tracking and behaviour analysis via XAI, ultimately aiding in the development of more efficient and targeted mosquito control measures, leading to significant public health benefits.

## Methods

A machine learning classifier has been established to classify male to non-male mosquitoes using 3D trajectories from mating swarms [18]. From this 3D dataset, corresponding 2D telecentric and 2D angular field of view information is derived to simulate the data obtained from these tracking systems. The sections below detail how the single-camera 2D telecentric and 2D angular field-of-view trajectories are determined and the corresponding features derived for the 2D data.

### Dataset description

The trajectories of the mosquitoes utilised in this investigation were produced by Butail et al. and were provided as 3D tracks following the processing steps outlined in [8]. The data were collected in Doneguebogou, Mali, for the years 2009–2011, during which wild *Anopheles gambiae* mosquito swarms were observed.

The dataset contained 191 male mosquito tracks over 12 experiments as well as 743 mating couple tracks (where male and female mosquitoes mate in flight and are tracked together) over 10 experiments (Table 1). The male mosquito tracks were captured in swarms where no females were present, whereas couple tracks were generated from swarms that contained mating events. Prior to analysis, tracks were filtered based on duration, excluding those < 3 s. This decreased the size of the dataset but effectively eliminated tracks with low information content.

**Table 1:** Numbers of experiments and tracks for each class of mosquito

Mosquito class	Number of experiments	Number of tracks	Number of tracks after filtering
Male	12	191	158
Mating couple	10	743	102
Female	1	6	4
Focal-male	1	6	6

The experiments used to track the mosquitoes utilised a stereo-camera set up using phase-locked Hitachi KP-F120CL cameras at 25 frames per second. Each camera captured 10-bit images with a resolution of 1392 × 1040 pixels. On-site calibration of the cameras was performed using a checkerboard and the MATLAB Calibration Toolbox [21]. The relative orientation and position of the cameras were established through extrinsic calibration, which involved capturing images of a stationary checkerboard in multiple orientations and positions. The camera's height, azimuth, and inclination were recorded to establish a reference frame fixed to the ground.

### Two-dimensional projection of 3D trajectory data

To conduct a comparative analysis between 3D and 2D trajectories, two methods were employed to convert the 3D dataset to a 2D one.

1. The first involves the omission of depth information, resulting in the plane of view parallel to the camera (YZ for this dataset). This method emulates a well-calibrated 2D setup that uses telecentric imaging [19], i.e. the separation of the two lenses on the imaging side generates the telecentric condition and any lens distortion effects have been removed by appropriate calibration.
2. The second transformation method utilises a single lens camera model placed a distance away from the swarm to project the trajectories onto a 2D plane (the camera detector plane), simulating the transformation that occurs through a single-camera setup including perspective and lens distortion.

To perform the second transformation, the camera was modelled using OpenCV [22] requiring focal length, principal points, distortion coefficients, and the camera location and rotation. The 3D trajectories were projected onto the image plane using a perspective transformation, utilising the projectPoints function (https://docs.opencv.org/4.x/d9/d0c/group__calib3d.html), represented by the distortion-free projection equation (Eq. 1):

1 $$\begin{array}{c}sp=A\left[R|t\right]{P}_{w}\end{array}$$

where ${P}_{w}$ is a four-element column vector in 3D homogeneous coordinates representing a point in the world coordinate system, $$p={\left[\begin{array}{ccc}u& v& 1\end{array}\right]}^{T}$$ is a three-element column vector in 2D homogeneous coordinates defining the corresponding position $\left(u,v\right)$ of a pixel in the image plane, $R$ and $t$ refer to the rotation and translation transformations between the world and camera coordinate systems, $s$ is a scaling factor independent of the camera model, and $A$ is the camera intrinsic matrix given by (Eq. 2).

2 $$\begin{array}{c}A=\left[\begin{array}{lll}{f}_{x}& 0& {c}_{x}\\ 0& {f}_{y}& {c}_{y}\\ 0& 0& 1\end{array}\right]\end{array}$$

with ${f}_{x}$ and ${f}_{y}$ the focal lengths expressed in pixel units, and ${c}_{x}$ and ${c}_{y}$ are the principal points on the detector in pixel units. Under these definitions the coordinates of the imaged point on the camera $\left(u,v\right)$ are in pixels. Radial, tangential, and prism distortions are included by modifying the 3D point in camera coordinates, given by $\left[R|t\right]{P}_{w}$ [22]. The camera intrinsic matrix values and distortion coefficients were based on the specifications provided by one of the camera models employed during the dataset generation process. These include the focal lengths (${f}_{x}=1993.208$ and ${f}_{y}=1986.203$), principal points (${c}_{x}=705.234$ and ${c}_{y}=515.751$) and distortion coefficients (${k}_{1}=-0.088547$, ${k}_{2}=0.292341$, and ${p}_{1}={p}_{2}=0$) [8]. To ensure accurate representation of the swarm, the translation vector was adjusted such that the optical axis aligns with the centre of a cuboid enclosing the swarm, while the camera model was positioned at a predetermined distance from the swarm centre. As detailed by Butail et al. [8], the camera was positioned between 1.5 m and 2.5 m away from the swarm. Therefore, in our simulated experiment, the camera model was positioned at 2 m from the swarm centre. Simulations were conducted with and without the lens distortion terms which showed that the vast majority (> 98%) of the distortion observed in the image was due to perspective at this range (for the camera intrinsic matrix values given above and a cuboid object extending 1 m in each axis). For the single-lens 2D camera model, the coordinates of the image points in pixels (from Eq. 1, corresponding to the 3D trajectory coordinates) were used directly for feature calculation and classification.

To investigate the impact of different distances between the camera and the swarm on classifier performance from a single-lens 2D measurement, adjustments in the focal length of the camera model were accounted for such that the swarm occupied the same extent in the image. The thin lens equation (Eq. 3) was used to approximate the distance from the lens to the image plane as the object distance is varied. This equation relates the focal length, $f$, to the distance of the object to the camera lens, $u$, and the distance of the camera lens to the image plane, $v$. Subsequently, by applying the magnification equation (Eq. 4), the magnification factor, $M$ , was determined [23]. Based on the new distance between the object and the lens, the corresponding focal length was calculated and utilised in the camera intrinsic matrix (Eq. 2).

3 $$\begin{array}{c}\frac{1}{f}=\frac{1}{u}+\frac{1}{v}\end{array}$$

4 $$\begin{array}{c}M=\frac{v}{u}\end{array}$$

**Fig. 4:**
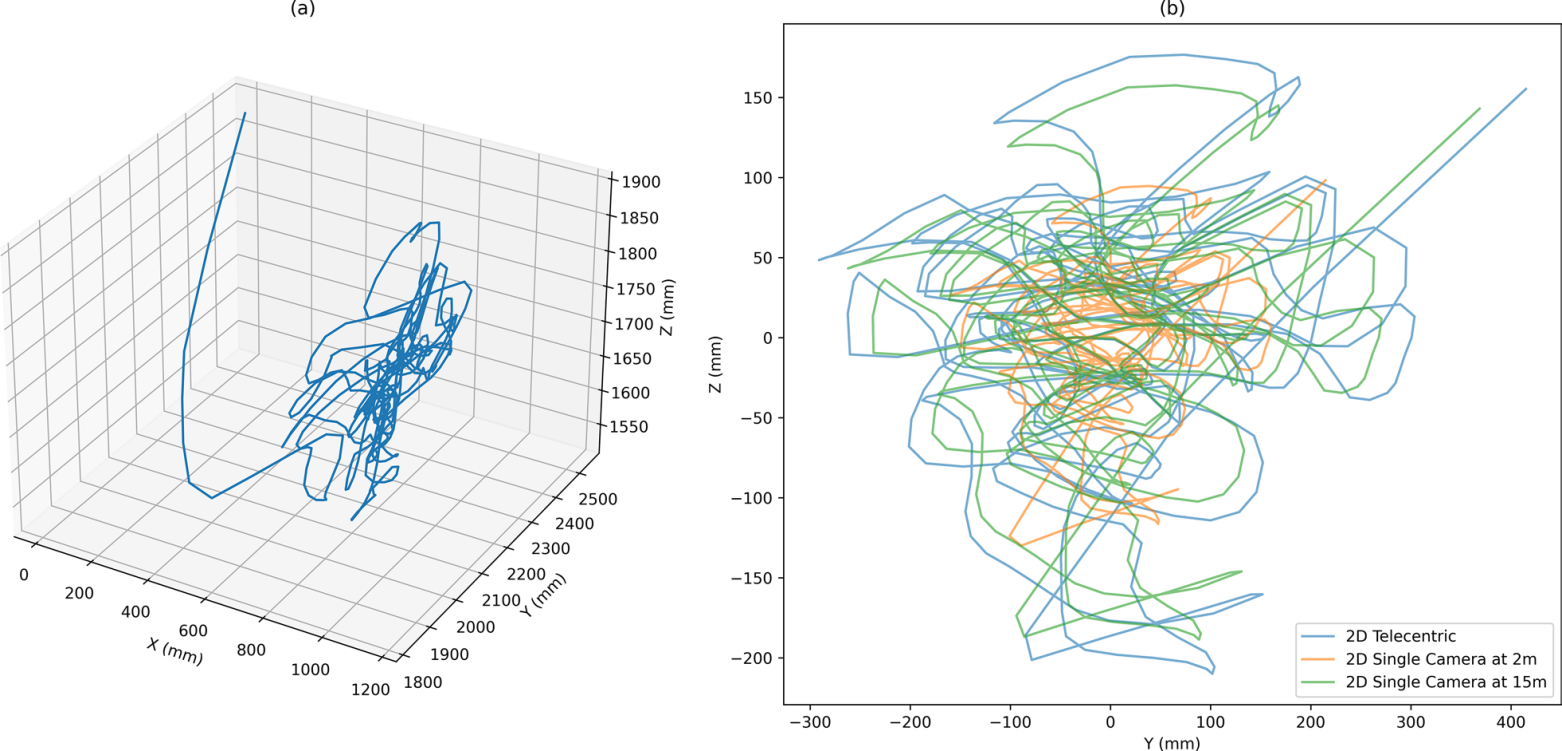
Plots displaying the effect of the transformation methods on a single mosquito trajectory. **a** Original 3D track. **b** Two transformation methods applied to the trajectory: the 2D telecentric transformation with depth information ignored (blue) and the 2D camera model developed in OpenCV at 2 m (orange) and 15 m (green), respectively, whilst utilising the distortion coefficients from Butail et al. [8]

Thereby, datasets for 3D, 2D telecentric, and 2D single camera at varying object distances were derived; an example is provided (Fig. 4).

### Machine learning framework

**Fig. 5:**
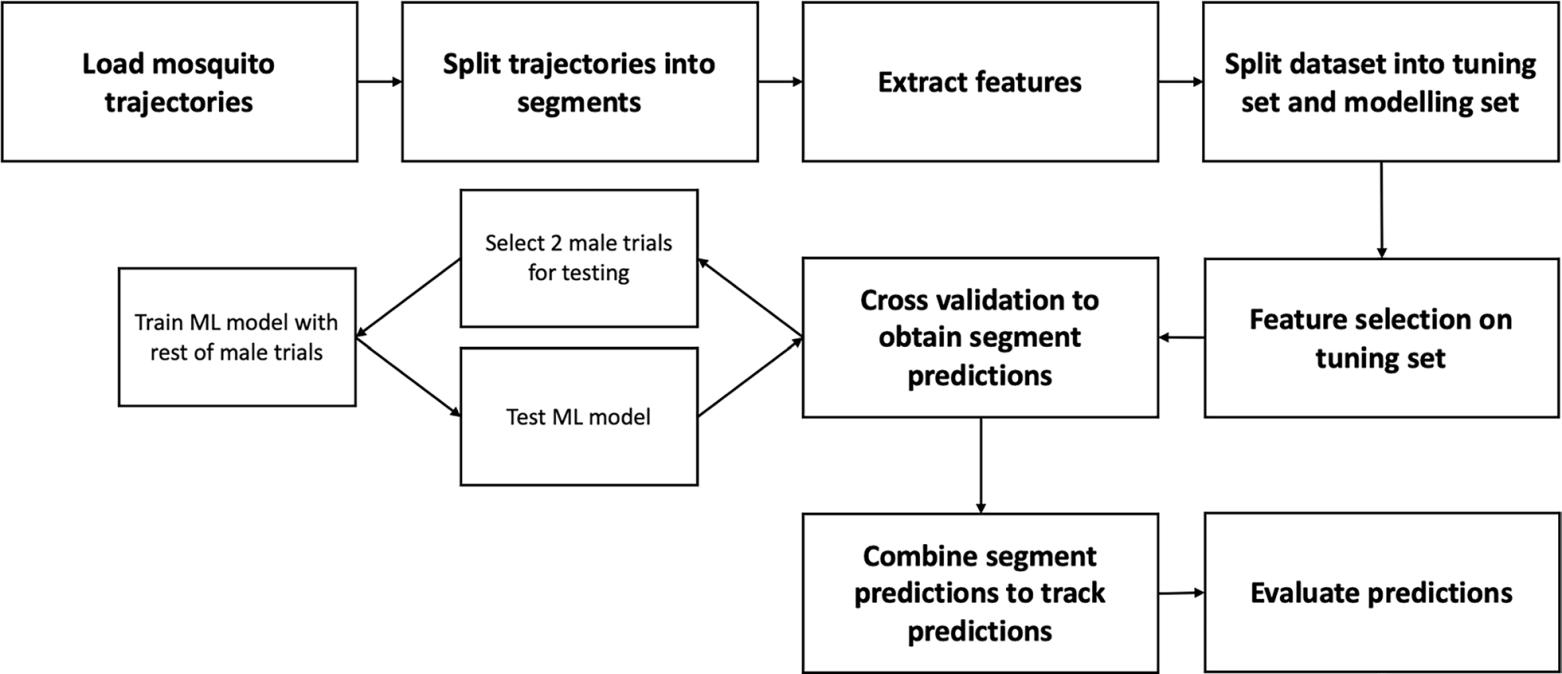
Diagram outlining the machine learning pipeline used to classify male and non-male mosquito tracks

This study employs an anomaly detection framework, as detailed in [18], to classify male and non-male mosquito tracks. Track durations are unified by splitting them into segments of equal duration, and flight features are extracted per segment. In [18], tracks shorter than double the segment length were removed. However, this restriction is removed as filtering was unified to remove tracks < 3 s in duration. This unifies the datasets and makes downstream comparison like-for-like. Features are selected using the Mann-Whitney U test and highly correlated features are removed. Classification is performed using a one-class support vector machine (SVM) model trained on a subset of the male class. The model forms predictions on track segments, and then a voting method is employed to return the final class prediction of whole tracks (Fig. 5).

The 3D trajectory feature set is detailed in [18]. For 2D trajectory data, an equivalent feature set was employed resulting in 136 features of flight, with most feature calculations remaining consistent, albeit with the exclusion of the third axis. For instance, straightness (also referred to as tortuosity) is computed as the ratio between the actual distance travelled and the shortest path between the start and end positions. For 3D trajectories, this was calculated as:

5 $$\begin{array}{c}S=\frac{{\sum }_{i=0}^{N}\sqrt{{\left({x}_{i+1}-{x}_{i}\right)}^{2}+{\left({y}_{i+1}-{y}_{i}\right)}^{2}+{\left({z}_{i+1}-{z}_{i}\right)}^{2}}}{\sqrt{{\left({x}_{N}-{x}_{0}\right)}^{2}+{\left({y}_{N}-{y}_{0}\right)}^{2}+{\left({z}_{N}-{z}_{0}\right)}^{2}}}\end{array}$$

However, in the 2D trajectory case, this was now calculated as:

6 $$\begin{array}{c}S=\frac{{\sum }_{i=0}^{N}\sqrt{{\left({x}_{i+1}-{x}_{i}\right)}^{2}+{\left({y}_{i+1}-{y}_{i}\right)}^{2}}}{\sqrt{{\left({x}_{N}-{x}_{0}\right)}^{2}+{\left({y}_{N}-{y}_{0}\right)}^{2}}}\end{array}$$

The calculations for the remaining features were originally devised for 2D trajectories. However, in the context of 3D trajectories, projections onto the X–Y, Y–Z, and X–Z planes were computed, resulting in the derivation of a single value. An example of this is the calculation of curvature, which requires a single plane:

7 $$\begin{array}{c}{k}_{i}=\frac{\dot{{x}_{i}}\ddot{{y}_{i}}-\dot{{y}_{i}}\ddot{{x}_{i}}}{{\left({\dot{{x}_{i}}}^{2}+{\dot{{y}_{i}}}^{2}\right)}^\frac{3}{2}}\end{array}$$

The study employed K-fold cross-validation. Two male trials were reserved for testing, while the remaining trials were used in training. All remaining classes (couples, females, and focal males) were used in testing. In the K-fold cross-validation process, different combinations of male trials were systematically rotated into the training set in each iteration, which is referred to as a ‘fold’. Performance metrics such as balanced accuracy, ROC AUC (area under the receiver operator curve), precision, recall, and F1 score were calculated with males and non-males considered as the positive class for metric computation.

The framework had various parameters that can be tuned including the machine learning model hyperparameters and the window size used to split tracks into segments. These were tuned together in a cross-validated grid search attempting to maximise balanced accuracy. An independent tuning set containing three male trials and two couple trials, distinct from the dataset used to report the classification performance and named the modelling set, was used to obtain the best parameters. The grid search utilised in this study encompassed a more refined range of values with a smaller step size compared to [18], which is detailed in the supplementary material. The hyperparameter, ν, described as “an upper bound on the fraction of training errors and a lower bound of the fraction of support vectors”, was set to 0.2. This value was chosen to make strong regularisation of the model to allow large errors on the male class (the only class that is seen during training) to reduce overfitting.

### Evaluation of transformed data

Various methods were used to assess the different datasets. The machine learning pipeline provides quantitative metrics for evaluating performance on the 3D and 2D trajectory feature sets. Analysing feature correlations between 3D/2D datasets can reveal insights into the preservation of flight features within 2D trajectories. Correlations were computed by calculating the average absolute Pearson’s correlation coefficient across features between two datasets. Even though each dataset has specific window parameters that are identified during hyperparameter tuning, a fixed segment size and overlap were used to determine the correlation matrix to generate paired samples.

An alternative technique for analysing and comparing features is to visualise them through an embedding. Here, an embedding is a lower dimensional space that condenses the information content from a higher dimensional space. Uniform manifold approximation and projection (UMAP) [24] creates a visualisation that shows how the 2D/3D datasets cluster within the embedded feature space. Notably, UMAP is a dimensionality reduction technique that preserves the local relationships and global structure of the data, making it particularly suitable for this purpose.

Most importantly, it is necessary to deduce whether the machine learning models are utilising features correctly and behavioural insights gathered are consistent with those from 3D trajectories. By using SHapley Additive exPlanations (SHAP) values [25], it was possible to visualise and explain how the model made its predictions.

From [18], classification of male and non-male trajectories based on 3D trajectory features was demonstrated, alongside XAI to interpret the machine learning model. The SHAP plots have increased noise due to using field data and may exhibit a slight skew in the colour scale. To ensure robust interpretations, SHAP scatter plots were also used to visualise the SHAP value distribution as a function of feature value.

## Results

**Fig. 6:**
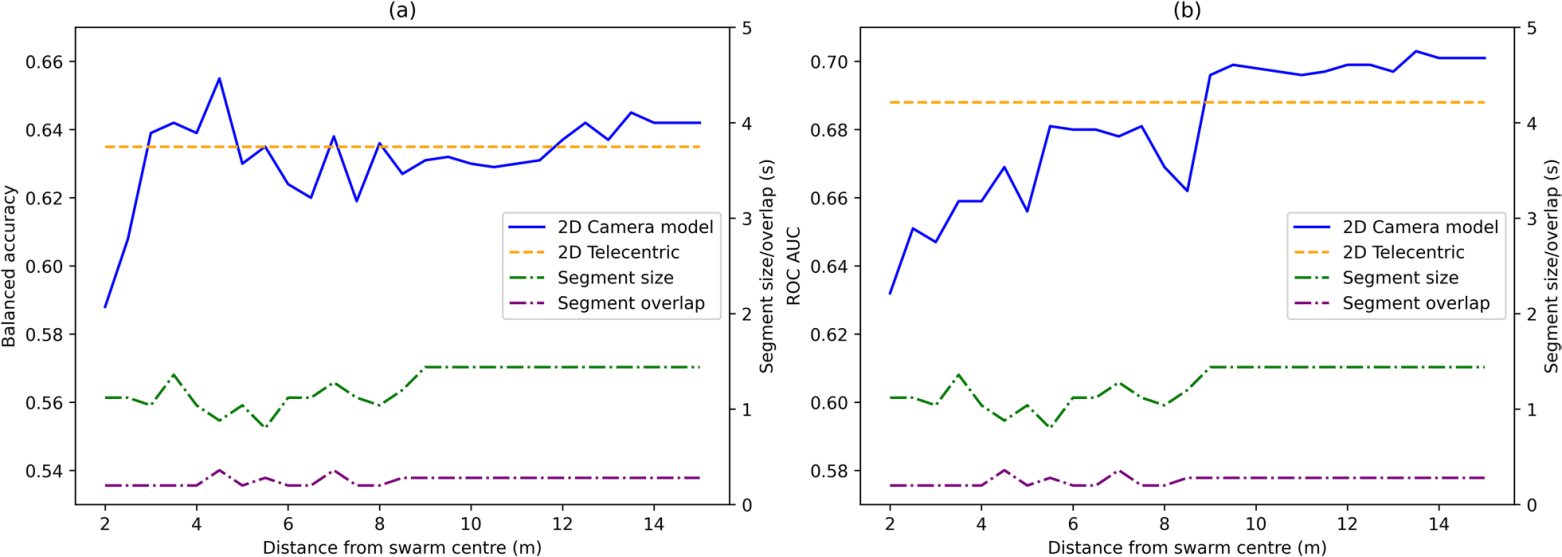
Classification performance of the 2D single-camera model as the distance varies. Solid lines: 2D single-camera model; dashed lines: data from 2D telecentric model. **a** Balanced accuracy as distance varies. **b** ROC AUC score as distance varies. Both graphs also display the optimised segment size and overlap from hyperparameter tuning at each distance

The 3D dataset was transformed into 2D telecentric and single-camera datasets at various distances from the swarm. Evaluating the machine learning framework’s performance at these distances (Fig. 6), the single-camera model closely matches the telecentric dataset as the camera moves farther from the object. For each distance, the tuned pipeline returns differing segment sizes and overlaps, which are also displayed.

Comprehensive results for the performance when using tuned pipeline parameters of the 3D dataset, 2D telecentric dataset, and 2D single-camera datasets with the camera placed at 2 m and 15 m are provided (Table 2). Across all datasets, the best performance obtained was from the 3D tracks with a balanced accuracy and ROC AUC score of 0.656 and 0.701. This performance may seem low, but the classifier is attempting to distinguish small differences in features of flight over segments of a few seconds and the data were captured in the field under various conditions; hence, such performance in this application is notable. Generally, the single-camera model performs worse than the telecentric and 3D methods in both cases. At 2 m, the single-camera model fares 6.8% and 6.9% worse in balanced accuracy and ROC AUC compared to the 3D dataset, primarily because of perspective distortion. The telecentric and 3D methods exhibit similar performance with absolute percentage differences of 2.1% and 1.0% in balanced accuracy and ROC AUC, respectively. This indicates preserved tracking accuracy with a 2D telecentric dataset, i.e. two orthogonal displacement components quantified, despite a loss of depth information. Similarly, when the single-camera model is placed farther away, its performance closely mirrors that of the 2D telecentric dataset with absolute percentage differences of 0.7% and 1.3% for balanced accuracy and ROC AUC respectively. Note that the performance metrics for the female and focal male classes are not conclusive as these results are based on a limited number of tracks.

**Table 2:** Performance metrics of each 3D/2D dataset when passed into the machine learning pipeline with the 95% confidence interval provided in brackets

	3D data	2D telecentric	2D single camera (at 2 m)	2D single camera (at 15 m)
Training set accuracy (male)	0.776 (0.733–0.821)	0.820 (0.750–0.896)	0.891 (0.837–0.950)	0.825 (0.761–0.875)
Testing set accuracy (male)	0.636 (0.270–0.937)	0.708 (0.279–0.983)	0.704 (0.277–0.971)	0.713 (0.279–0.970)
Testing set accuracy (couple)	0.627 (0.594–0.674)	0.518 (0.406–0.594)	0.441 (0.348–0.565)	0.533 (0.442–0.630)
Testing set accuracy (female)	1.000 (1.000–1.000)	1.000 (1.000–1.000)	0.750 (0.750–0.750)	0.929 (0.750–1.000)
Testing set accuracy (focal male)	0.778 (0.583–0.833)	0.786 (0.667–0.833)	0.786 (0.667–0.833)	0.786 (0.667–0.833)
Balanced accuracy	0.656 (0.506–0.776)	0.635 (0.484–0.709)	0.588 (0.428–0.690)	0.642 (0.495–0.725)
ROC AUC	0.701 (0.618–0.763)	0.688 (0.543–0.805)	0.632 (0.452–0.774)	0.701 (0.550–0.808)
F1 (average)	0.635 (0.501–0.734)	0.597 (0.475–0.654)	0.537 (0.426–0.640)	0.604 (0.485–0.687)
F1 (male as positive class)	0.555 (0.334–0.718)	0.546 (0.317–0.672)	0.505 (0.313–0.641)	0.552 (0.323–0.695)
F1 (nonmale as positive class)	0.715 (0.662–0.756)	0.647 (0.601–0.677)	0.569 (0.525–0.642)	0.656 (0.607–0.699)
Recall (average)	0.656 (0.506–0.776)	0.635 (0.484–0.709)	0.588 (0.428–0.690)	0.642 (0.495–0.725)
Recall (male as positive class)	0.664 (0.381–0.921)	0.725 (0.386–0.961)	0.718 (0.363–0.939)	0.730 (0.386–0.95)
Recall (nonmale as positive class)	0.648 (0.616–0.692)	0.545 (0.438–0.616)	0.458 (0.370–0.575)	0.554 (0.459–0.651)
Precision (average)	0.642 (0.504–0.759)	0.629 (0.483–0.722)	0.587 (0.435–0.691)	0.635 (0.492–0.738)
Precision (male as positive class)	0.481 (0.299–0.606)	0.442 (0.271–0.538)	0.397 (0.275–0.514)	0.449 (0.281–0.568)
Precision (nonmale as positive class)	0.803 (0.665–0.931)	0.816 (0.676–0.956)	0.778 (0.595–0.937)	0.821 (0.677–0.944)

A closer analysis of individual fold performance across the datasets revealed additional understanding. As reported in [18], poorly performing folds were those that were tested on abnormal trials where mosquito type was different (Mopti form instead of Savannah form) and swarm location differed (over bundles of wood rather than bare ground). These conditions could alter mosquito trajectory features, potentially causing them to fall outside the decision boundary of the single-class model. Conversely, folds including abnormal trials in training consistently performed best. This indicates potential overfitting to the variability within their features, leading to accurate classifications for male mosquitoes but reduced accuracy for non-male. This trend held for both the 2D telecentric and single-camera models at 15 m. However, the 2 m single-camera model displays the opposite behaviour, with the best performance on folds containing abnormal trials in testing. This implies that the perspective distortion introduced by the camera at this distance is affecting the feature values and their variability, resulting in unexpected performance variations across different trials.

**Fig. 7:**
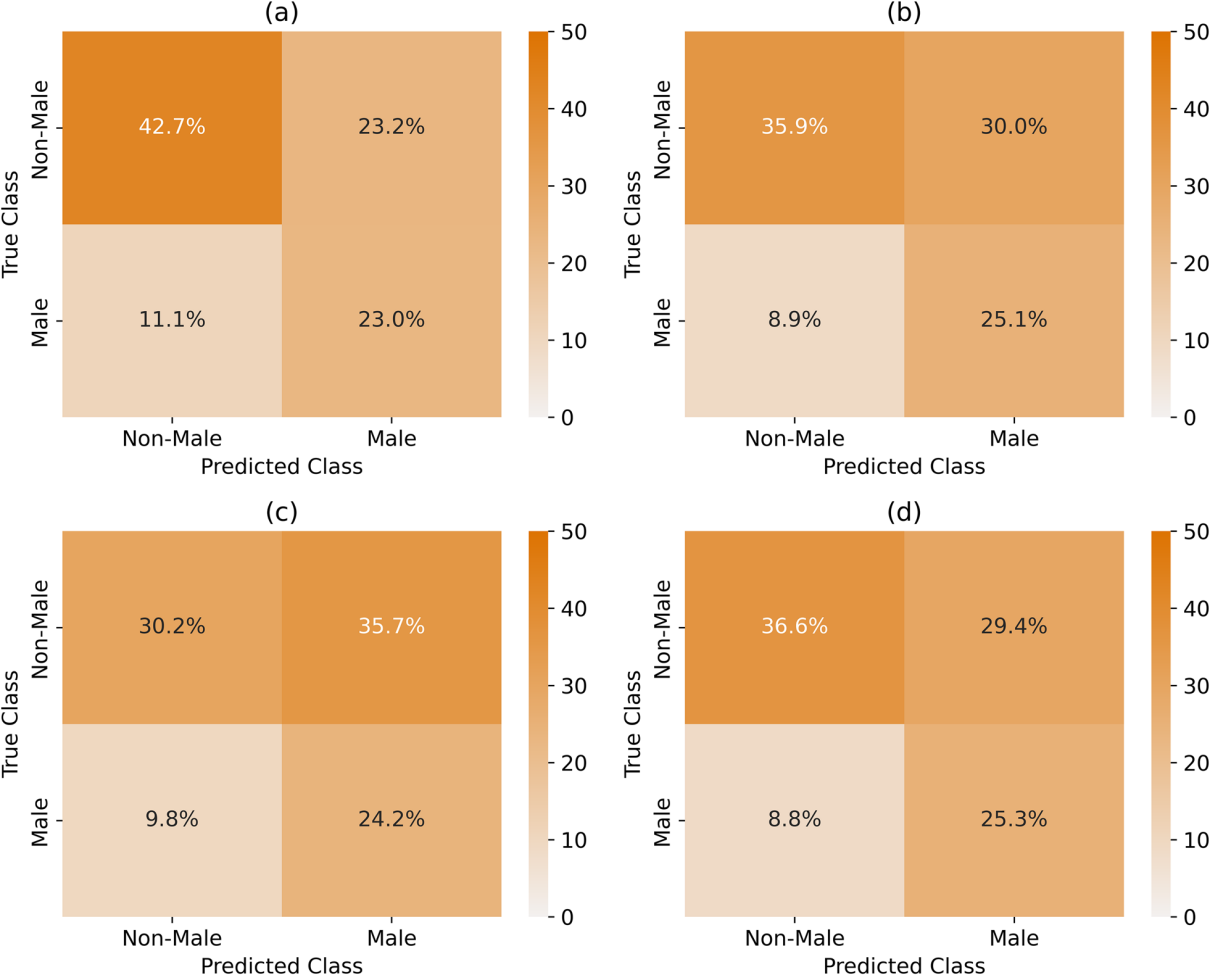
Confusion matrices of each dataset: **a** original 3D dataset, **b** 2D telecentric dataset, **c** 2D single-camera model at 2 m, and **d** 2D single-camera model at 15 m

**Fig. 8:**
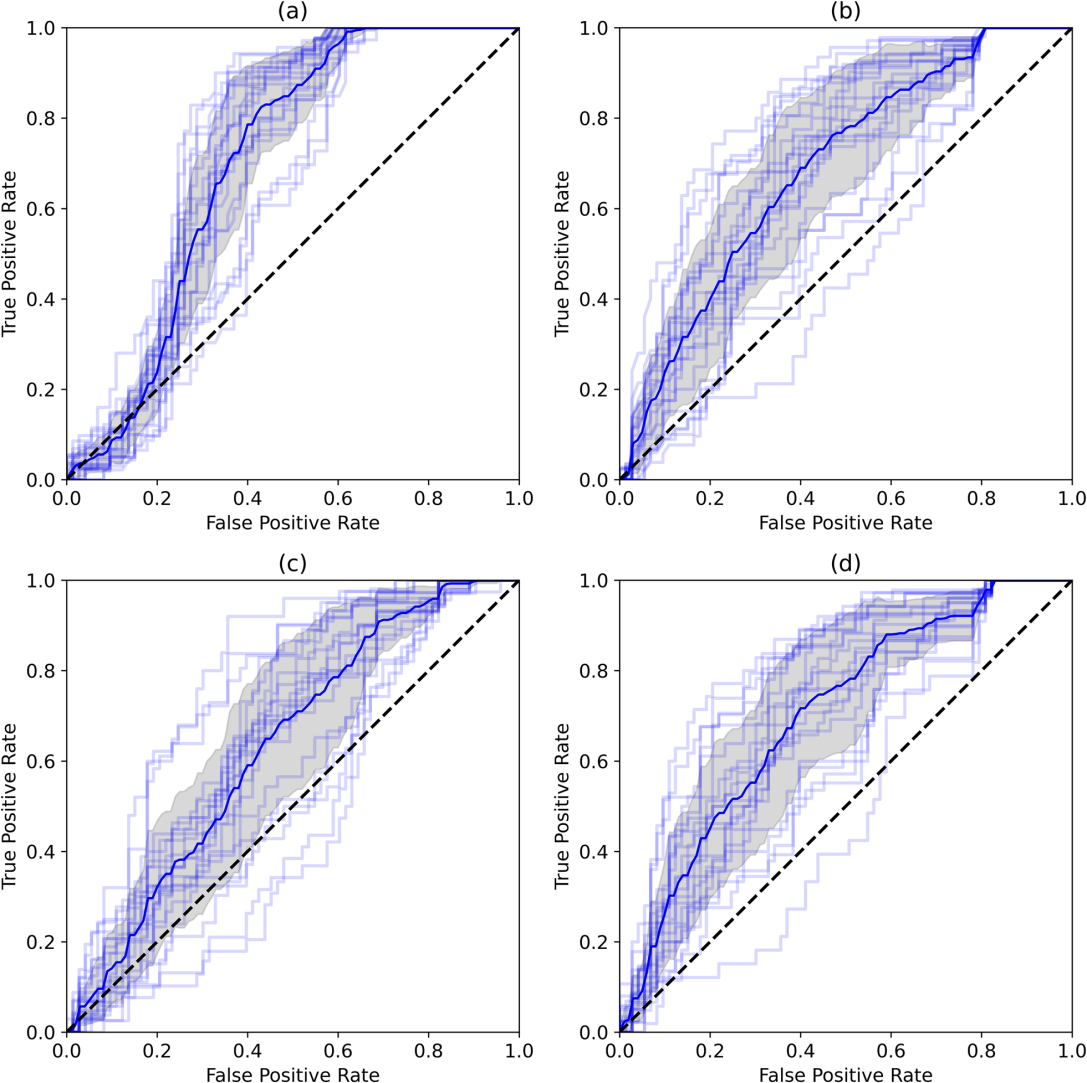
Receiver-operator characteristic (ROC) curves of each dataset. The dark blue line displays the average ROC curve across all folds, the light blue lines show the ROC curve at each fold and the grey shadow depicts the standard deviation. Within the figure, (**a**) displays the original 3D dataset, (**b**) the 2D telecentric dataset, (**c**) the 2D single-camera model at 2 m, and (**d**) 2D single-camera model at 15 m

The performance of these models can be visualised through confusion matrices (Fig. 7) and receiver-operator characteristic (ROC) curves (Fig. 8). The confusion matrices display the predictions of all folds with the percentage of predictions labelled in each section of the matrix. The ROC curves depict the performance of a binary classifier by plotting the trade-off between true- and false-positive rate.

**Fig. 9:**
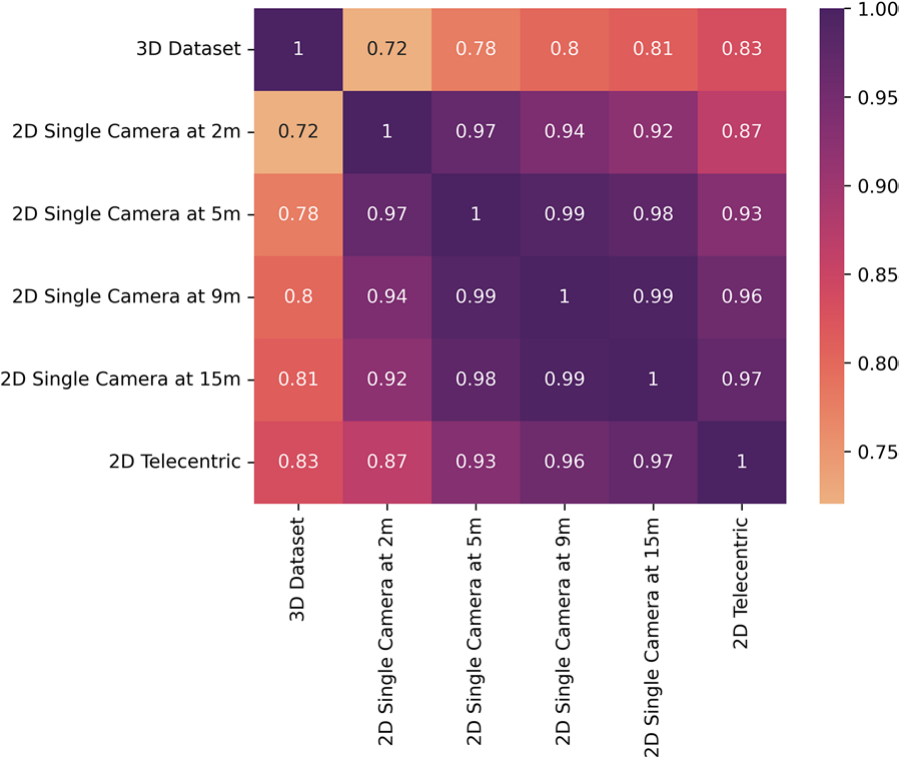
Pairwise correlation matrix between each dataset. The Pearson correlation between the same features for each pair of datasets is computed, with the average of the correlations taken to return a final value for the dataset pairs

Analysing the correlation between features from different datasets can reveal insights into the preservation of flight features in 2D trajectories. Datasets were generated at various distances using the same segment size and overlap, such that correlation can be computed between paired samples. To compute the correlation, each of the datasets was pairwise correlated to produce the matrix (Fig. 9). Overall, utilising a 2D telecentric setup preserves more features compared to the 3D dataset, with an average correlation of 0.83. Shape descriptors show the lowest correlation because of depth loss, which is expected. Conversely, a single-camera setup compromises tracking accuracy, resulting in lower feature correlation compared to a 3D stereoscopic system. The average correlation between the 2D single camera at 2 m with the 3D dataset and the 2D telecentric system is 0.72 and 0.87, respectively. However, positioning the single camera at 9 m significantly improves correlation. Average correlation values increase to 0.80 and 0.96 compared to 3D and 2D telecentric datasets, respectively. These results are expected as increasing camera-swarm distance reduces the perspective distortion effect, thereby resembling telecentric setup data and enhances feature preservation.

**Fig. 10:**
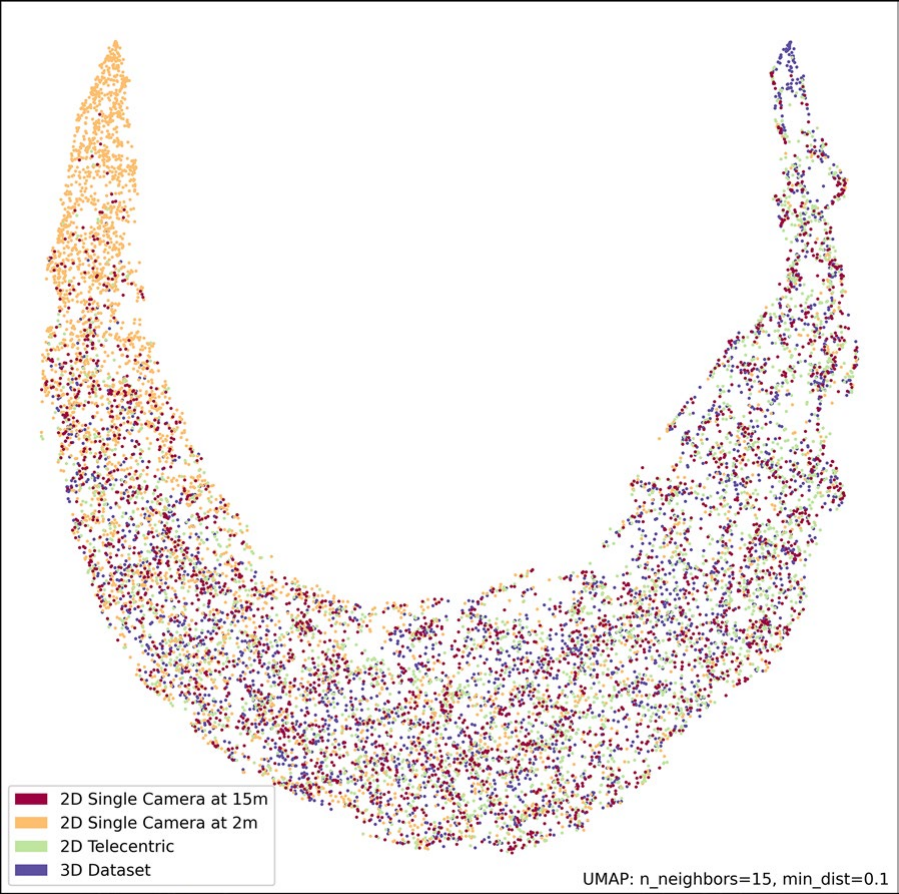
UMAP representation of each of the datasets

**Fig. 11:**
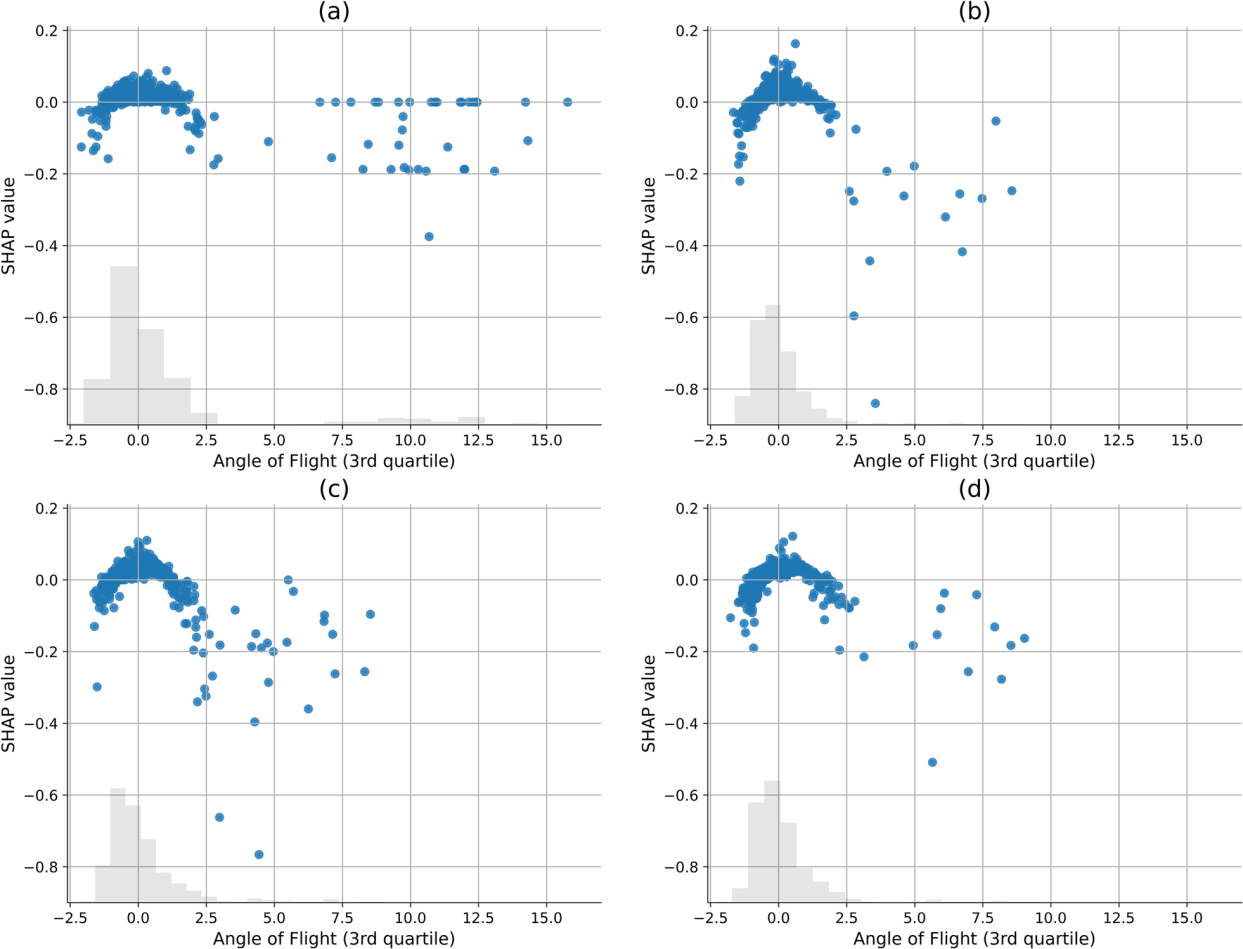
SHAP scatter plots for the third quartile of angle of flight feature. Within the figure, (**a**) displays the original 3D dataset, (**b**) the 2D telecentric dataset, (**c**) the 2D single-camera model at 2 m, and (**d**) 2D single-camera model at 15 m

The UMAP representation (Fig. 10) provides a clear visualisation of the disparities between the datasets. The SHAP plots of the best performing folds for each model were generated and are provided in the supplementary material. This includes SHAP summary plots for the best performing folds for the 3D, 2D telecentric, and 2D single-camera model at 2 m and 2D single camera model at 15 m datasets, respectively (Additional file 1: Figs. S1-S4). The supplementary material also includes SHAP summary plots where only the common features across each model are selected and sorted alphabetically (Additional file 1: Figs. S5–S8). SHAP scatter plots for the third quartile of angle of flight feature are provided for each dataset (Fig. 11). This feature was chosen as an example to illustrate the impact that each camera system has on SHAP and feature values. In this figure, each point represents a segment, with its corresponding normalised feature value on the x-axis and its SHAP value on the y-axis. A histogram of the segment feature values is provided as a grey shadow.

The feature selection process for each dataset selects slightly different types of features. Among the datasets, the numbers of selected features are as follows: 61 for the 3D dataset, 34 for the 2D telecentric dataset, 42 for the 2D single-camera dataset at 2 m, and 35 for the 2D single-camera dataset at 15 m. Notably, the 3D dataset contains more features as it includes some feature calculations projected in the X–Y, Y–Z,, and X–Z planes which are not present with 2D data. Despite these differences, a significant portion of features is shared between them. Specifically, 85% of the features are common between the 2D telecentric and 2D single-camera datasets at 2 m, while 97% of the features are common between the 2D telecentric and 2D single-camera datasets at 15 m. These observations further reaffirm that the 2D single-camera dataset at 15 m can effectively emulate a 2D telecentric system. It is important to note that across all datasets, only a few shape descriptors are selected, consistent with the findings from [18].

## Discussion

This study compares 3D and 2D trajectory datasets simulating various imaging techniques. Performance metrics were obtained via a one-class machine learning classifier on field data of male and non-male mosquitoes in a mating swarm. Generally, the 3D and 2D telecentric datasets performed best, with the exception of some metrics from the 2D single-camera model at 15 m. Performance with a single camera at a great distance (with a suitable focal length lens) approached that of the telecentric dataset. However, at a typical distance for insect tracking of around 2 m, performance showed an average decrease of about 0.05 across all metrics on the test datasets.

Earlier, we hypothesised that 2D telecentric imaging data would perform similarly to stereoscopic 3D data despite the loss of one axis of information. We anticipated that a single-camera model would be less effective at short distances compared to larger distances, where trajectory data align more closely with telecentric imaging (with larger focal length imaging lenses). The machine learning classifier performance metrics confirm both hypotheses. The implication of the first hypothesis is that the necessary features to differentiate the behaviour of male compared to non-male mosquitoes are present in two orthogonal components of motion as well as in a complete three-dimensional measurement. This is different from what we normally consider to be the accuracy of a measurement. In terms of metrology, accuracy is the difference between a measurement and the true value. The speed of a mosquito requires all three velocity components for accurate determination. The findings demonstrate that features extracted from 2D orthogonal, i.e. independent axes, measurements can characterise behaviour comparably to 3D measurements (Table 2).

Single-camera 2D data are typically obtained without calibrating for geometric distortions introduced by the imaging lens. Distortion increases linearly with radial distance from the optical axis and is a power law with respect to numerical aperture [26] and the angular field of view increases as the camera is moved closer to the scene of interest. Hence, close range imaging yields higher distortion compared to distant imaging for the same field of view. Perspective effects at close range mean that the two components of position measured at a detector are also a function of object position along the optical axis and the magnitude increases with radial distance (as for lens distortion). Hence, it appears that classifier performance is impacted by perspective and distortion aberrations, particularly noticeable at closer distances. Conversely, positioning the camera further away reduces perspective distortion, leading to more reliable interpretations akin to 2D telecentric data. However, by tuning the parameters for the machine learning pipeline at each distance, the pipeline partially accommodates for the distortion effects introduced at smaller distances. The changing segment size for each distance, as determined by the tuning dataset, thus plays a strong role in the classification performance leading to some of the variations in balanced accuracy and ROC AUC as distance increases. The figure (Fig. 6) captures these variations and also depicts the tuned segment size and overlap at each distance. Differing segment sizes capture different scales of behaviour and would lead to variations in feature values and thus differences in classification performance. Intriguingly, this study found that achieving comparable performance between a single-camera 2D measurement and the corresponding 2D telecentric assessment occurs at a range of 9–10 m. The pipeline parameters after 9 m remain consistent and are equivalent to the telecentric system, displaying its effectiveness at emulating a telecentric camera system.

The correlation analysis highlights differences between the camera systems. The single-camera model at 15 m correlates strongly with 2D telecentric data (0.96), while 2D telecentric data correlate well with the 3D dataset (0.83). In the UMAP representation (Fig. 10), features from the 2D single camera at 2 m cluster towards the upper left corner, suggesting less reliable and inconsistent object tracking at close range. SHAP scatter plots for the angle of flight, third quartile, feature (Fig. 11) corresponding to the four different imaging setups demonstrate similarity among the 3D, telecentric, and single camera at 15 m, whereas the single camera at 2 m has increased noise and overlap between the classes across some feature values. This feature describes the upper quartile of the change in angle of flight distribution within a track segment, where high values indicate a large deviation. It can be argued that this feature for the 3D dataset shows the clearest separation between the male and non-male classes, while overlap occurs in other setups. In the single camera at 2 m SHAP scatter plot, the histogram displays a distorted distribution of normalised feature values compared to the other histograms, further illustrating the impact the distortion that camera systems at close distance bring. SHAP summary plots in the supplementary information confirm these trends indicating subtle differences in feature contributions and a slight skew towards male predictions with close-range single-camera models. This phenomenon can be attributed to the perspective distortion introduced in trajectories that are constructed by a single-camera model, resulting in highly variable features across all classes. Consequently, the distinct separation between classes diminishes for 2D imaging at close range.

The study primarily focused on the Y–Z view directly imaged on the camera detector, but the other two orthogonal views were assessed (Additional file 1: Figs. S12-S13). Notably, the machine learning model performance of these additional views were higher than that of the original view that has been discussed. Specifically, the overhead view X–Y, which captures the distinctive circular motions of swarming male mosquitoes and the more erratic behaviours of mating couples, likely contributed to its higher effectiveness. The X–Z plane, observing the swarm from the other side view, may perform better because of the increased uncertainty of X-positional data in combination with perspective, which may amplify the depth information (e.g. through increased variability in certain features). Mating couple tracks move less in the depth plane and thus lead to bias towards one of the classes. Both these views utilise the depth axis that, while derived, introduces significant noise, rendering these findings less reliable. During the generation of the dataset, the camera system is placed 1.5–2.5 m away from the swarm and the baseline is 20 cm [8], meaning the angle subtended by the cameras at the swarm in the stereoscopic setup varies between 4.6 and 7.6 degrees. According to [27] for a related stereoscopic imaging setup, with an angle of 5 degrees, the uncertainty in depth displacements is > 11 times the uncertainty parallel to the detector plane. With an angle of 7.5 degrees, this uncertainty is > 7 times the uncertainty parallel to the detector plane. As a result, the accuracy of the depth component (X) is 7–11 times worse than the other measurement components, and thus these results from the other views are unreliable.

Overall, the 3D dataset demonstrates superior performance, followed by the telecentric dataset. Both setups can be configured in a small experimental footprint compatible with experimental hut trials in sub-Saharan Africa. Stereo 3D setups require alignment of the two cameras on the same field of view and in situ calibration. Two-dimensional telecentric setups require large aperture optics typically achieved with plastic Fresnel lenses [19], the same size as the required field of view, and careful alignment of the separation between the camera and large aperture lenses. Single-camera 2D imaging is experimentally simpler and can be done with lower camera to object distance within the size of a typical experimental hut but then generate the distortions described above and lower performance in machine learning classification and hence difficulties in behaviour interpretation. Two-dimensional imaging at longer range becomes problematic for practical reasons, the image path would extend outside a typical dwelling, and it is difficult to prevent occlusion by people and animals during recordings that can take several hours. Also, with large focal length lenses, outdoor implementation in low light conditions can be particularly problematic as the optical efficiency reduces, an effect that has not been investigated here. It is also recognised that the calibration process for stereoscopic imaging naturally means that trajectories are obtained in physical distance units, e.g. mm; telecentric setups can also be relatively easily calibrated as position data parallel to the camera detector remains the same irrespective of an object’s position along the optical axis. The resulting machine learning models can therefore be applied to results of other, equally well calibrated experiments that attempt to elicit similar behaviours. Two-dimensional single-camera measurements are obtained in pixels from the detector—whilst known artefacts could be placed in the field of view for calibration, manual assessment of whether trajectories are in the appropriate depth plane would need to be made. Hence, the machine learning models from 2D single-camera measurements are less useful than the calibrated data from stereoscopic 3D or telecentric 2D setups.

There are certain limitations with this study that should be acknowledged. First, the datasets used for comparing the performance of different tracking systems were all simulated, except for the 3D dataset. The 3D data used for simulating the other tracking systems were gathered from mosquito swarms, where their movement revolves around a central point, resulting in generally symmetric trajectories (especially in both horizontal axes). As a result, these findings may not be applicable in studies that have unsymmetrical movements (e.g. mosquito flight around bednets [19]). The orientation of the 2D datasets is to primarily capture the vertical axis, with respect to the ground, and one horizontal axis. It is probably important for 2D datasets to include the effect of gravity and one other orthogonal axis. Were a trajectory to be along a linear axis not captured by a 2D imaging system, then clearly it would fail to provide useful information. However, the mating swarm data used here [8], data from field tests tracking mosquitoes around human baited insecticide treated nets [28] or in odour stimulated wind tunnels tests [29], mosquitoes do not exhibit straight line flight behaviours. The 3D data itself were gathered from wild mosquito swarms and as such the trajectories may already contain noise that may reduce performance across all tracking simulations. To further validate these findings, future trials of the various tracking systems should be tested by generating new experimental data from each system in diverse scenarios and then comparing their trajectories to determine whether the same behaviours and trends between the 3D and 2D datasets are observed.

## Conclusions

Accurately tracking mosquitoes, or more generally insects, is a difficult task that requires care to be taken at many stages. This includes considering the experimental conditions, the video recording equipment, and the software used to identify insects from videos. Nonetheless, accurate tracking of mosquitoes could lead towards improved understanding of their behaviours that may influence disease transmission intervention mechanisms. The results of this study imply that 2D telecentric and 3D stereoscopic imaging should be the preferred imaging approaches to adequately capture mosquito behaviour for machine learning analysis. Both of these approaches are compatible with laboratory and field-based studies, but it should be recognised that 2D telecentric imaging is less complex and the data more straightforward to process. Single-camera 2D imaging over large, metre-scale field of view, although experimentally easier and needing less expensive equipment, should be avoided because of the distortion in the results and subsequent difficulty in interpretation. Nonetheless, if a single camera is placed at a considerable distance from the object of interest, achieving accurate interpretations of behaviour may be feasible. However, this demands expensive long focus lenses and a strong light source to effectively record trackable mosquitoes.

### Supplementary Information

**Additional file 1: Figure S1.** SHAP summary plot for the best fold using the 3D dataset. **Figure S2.** SHAP summary plot for the best fold using the 3D dataset. **Figure S3**. SHAP summary plot for the best fold using the 3D dataset. **Figure S4.** SHAP summary plot for the best fold using the 2D single camera at 15 m dataset. **Figure S5.** SHAP summary plot of the best fold using the 3D dataset only selecting the common features across all datasets sorted alphabetically. **Figure S6**. SHAP summary plot of the best fold using the 2D telecentric dataset only selecting the common features across all datasets sorted alphabetically. **Figure S7**. SHAP summary plot of the best fold using the 2D single camera at 2 m dataset only selecting the common features across all datasets sorted alphabetically. **Figure S8**. SHAP summary plot of the best fold using the 2D single camera at 15 m dataset only selecting the common features across all datasets sorted alphabetically. **Table S9.** Features extracted and their corresponding statistical features from 2D data. **Table S10.** Hyperparameter tuning parameter ranges. **Table S11.** Selected hyperparameters for each dataset after tuning. **Figure S12.** Performance as distance increases for the X–Y plane (top view). (a) Displays balanced accuracy and (b) displays ROC AUC score. **Figure S13.** Performance as distance increases for the X–Z plane (other side view). (a) Displays balanced accuracy and (b) displays ROC AUC score.

## Data Availability

The data presented in this study are available on request from Butail et al. The code used in this paper has been deposited to and made publicly available from the authors’ GitHub repository, https://github.com/yasserqureshi1/double-vision.

## References

[1] Helmer J. Article [Internet]. Pfizer; 2016 [cited 2023 Jun 25]. Available from: https://www.pfizer.com/news/articles/mosquito_as_deadly_menace.

[2] Murray GP, Lissenden N, Jones J, Voloshin V, Toé KH, Sherrard-Smith E, et al. Barrier bednets target malaria vectors and expand the range of usable insecticides. Nat Microbiol. 2019;5:40–7. 10.1038/s41564-019-0607-2. 31792426 10.1038/s41564-019-0607-2

[3] Yee WL, Foster WA. Diel sugar-feeding and host-seeking rhythms in mosquitoes (Diptera: Culicidae) under laboratory conditions. J Med Entomol. 1992;29:784–91. 10.1093/jmedent/29.5.784. 1357175 10.1093/jmedent/29.5.784

[4] Benelli G. The best time to have sex: mating behaviour and effect of daylight time on male sexual competitiveness in the Asian tiger mosquito, Aedes albopictus (Diptera: Culicidae). Parasitol Res. 2014;114:887–94. 10.1007/s00436-014-4252-7. 25487029 10.1007/s00436-014-4252-7

[5] Healy TP, Copland MJ. Activation of anopheles gambiae mosquitoes by carbon dioxide and human breath. Med Vet Entomol. 1995;9:331–6. 10.1111/j.1365-2915.1995.tb00143.x. 7548953 10.1111/j.1365-2915.1995.tb00143.x

[6] Yao R, Lin G, Xia S, Zhao J, Zhou Y. Video object segmentation and tracking. ACM Trans Intel Syst Technol. 2020;11:1–47. 10.1145/3391743. 10.1145/3391743

[7] Stamou G, Krinidis M, Loutas E, Nikolaidis N, Pitas I. 2D and 3D motion tracking in Digital Video. Handbook of Image and Video Processing. 2005;491–517. 10.1016/b978-012119792-6/50093-0.

[8] Butail S, Manoukis N, Diallo M, Ribeiro JM, Lehmann T, Paley DA. Reconstructing the flight kinematics of swarming and mating in wild mosquitoes. J R Soc Interface. 2012;9:2624–38. 10.1098/rsif.2012.0150. 22628212 10.1098/rsif.2012.0150 PMC3427502

[9] Pérez-Escudero A, Vicente-Page J, Hinz RC, Arganda S, de Polavieja GG. IdTracker: tracking individuals in a group by automatic identification of unmarked animals. Nat Methods. 2014;11:743–8. 10.1038/nmeth.2994. 24880877 10.1038/nmeth.2994

[10] Mathis A, Mamidanna P, Cury KM, Abe T, Murthy VN, Mathis MW, et al. Deeplabcut: markerless pose estimation of user-defined body parts with deep learning. Nat Neurosci. 2018;2021:1281–9. 10.1038/s41593-018-0209-y. 10.1038/s41593-018-0209-y 30127430

[11] Pereira TD, Tabris N, Matsliah A, Turner DM, Li J, Ravindranath S, et al. Sleap: a deep learning system for multi-animal pose tracking. Nat Methods. 2022;19:486–95. 10.1038/s41592-022-01426-1. 35379947 10.1038/s41592-022-01426-1 PMC9007740

[12] Hollows G, James N. The advantages of Telecentricity [Internet]. 2015 [cited 2023 Nov 1]. Available from: https://www.edmundoptics.co.uk/knowledge-center/application-notes/imaging/advantages-of-telecentricity.

[13] Lange B. Fixed focal length or telecentric lens? Photonics Views. 2022;19:41–3. 10.1002/phvs.202200034. 10.1002/phvs.202200034

[14] Macrì S, Neri D, Ruberto T, Mwaffo V, Butail S, Porfiri M. Three-dimensional scoring of zebrafish behavior unveils biological phenomena hidden by two-dimensional analyses. Sci Rep. 2017.16;7(1). 10.1038/s41598-017-01990-z. 28512334 10.1038/s41598-017-01990-z PMC5434067

[15] Ladu F, Bartolini T, Panitz SG, Chiarotti F, Butail S, Macrì S, et al. Live predators, robots, and computer-animated images elicit differential avoidance responses in zebrafish. Zebrafish. 2015;12:205–14. 10.1089/zeb.2014.1041. 25734228 10.1089/zeb.2014.1041

[16] Fabian ST, Sondhi Y, Allen PE, Theobald JC, Lin H-T. Why flying insects gather at artificial light. Nat Commun. 2024. 30;15(1):689. 10.1038/s41467-024-44785-3. 38291028 10.1038/s41467-024-44785-3 PMC10827719

[17] Butail S, Manoukis NC, Diallo M, Ribeiro JM, Paley DA. The dance of male *Anopheles gambiae *in wild mating swarms. J Med Entomol. 2013;50:552–9. 10.1603/me12251. 23802449 10.1603/me12251 PMC4780853

[18] Qureshi YM, Voloshin V, Facchinelli L, McCall PJ, Chervova O, Towers CE, et al. Finding a husband: using explainable AI to define male mosquito flight differences. Biology. 2023;12:496. 10.3390/biology12040496. 37106697 10.3390/biology12040496 PMC10135534

[19] Parker JEA, Angarita-Jaimes N, Abe M, Towers CE, Towers D, McCall PJ. Infrared video tracking of anopheles gambiae at insecticide-treated bed nets reveals rapid decisive impact after brief localised net contact. Sci Rep. 2015. 1;5(1). 10.1038/srep13392. 26323965 10.1038/srep13392 PMC4642575

[20] Machraoui AN, Diouani MF, Mouelhi A, Jaouadi K, Ghrab J, Abdelmelek H, et al. Automatic identification and behavioral analysis of phlebotomine sand flies using trajectory features. Vis Comput. 2018;35:721–38. 10.1007/s00371-018-1506-x. 10.1007/s00371-018-1506-x

[21] Bouguet J. Matlab Camera Calibration Toolbox [Internet]. 2000 [cited 2023 Nov 1]. Available from: https://www.vision.caltech.edu/bouguetj/calib_doc/index.html.

[22] Pulli K, Baksheev A, Kornyakov K, Eruhimov V. Real-time computer vision with opencv. Commun ACM. 2012;55:61–9. 10.1145/2184319.2184337. 10.1145/2184319.2184337

[23] Lawrence RW. Magnification ratio and the lens equations. Phys Teacher. 2000;38:170–1. 10.1119/1.880487. 10.1119/1.880487

[24] McInnes L, Healy J, Saul N, Großberger L. UMAP: uniform manifold approximation and projection. J Open Source Softw. 2018;3:861. 10.21105/joss.00861. 10.21105/joss.00861

[25] Lundberg S, Lee SI. A unified approach to interpreting model predictions. In: Proceedings of the 31st Conference on Neural Information Processing Systems (NIPS); 2017 Dec 4–9; Long Beach, CA, USA. 2017. p. 4768–4777.

[26] Born M, Wolf E, Bhatia AB. Chapter 5—geometrical theory of aberrations. In Principles of Optics. Cambridge: Cambridge University Press; 2019. pp. 228–260.

[27] Lawson NJ, Wu J. Three-dimensional particle image velocimetry: error analysis of stereoscopic techniques. Meas Sci Technol. 1997;8:894–900. 10.1088/0957-0233/8/8/010. 10.1088/0957-0233/8/8/010

[28] Angarita-Jaimes NC, Parker JE, Abe M, Mashauri F, Martine J, Towers CE, et al. A novel video-tracking system to quantify the behaviour of nocturnal mosquitoes attacking human hosts in the field. J R Soc Interface. 2016;13:20150974. 10.1098/rsif.2015.0974. 27075002 10.1098/rsif.2015.0974 PMC4874425

[29] Spitzen J, Takken W, Pates Jamet HV, Ponzio C, Koenraadt CJ. Absence of close-range excitorepellent effects in malaria mosquitoes exposed to deltamethrin-treated bed nets. Am J Trop Med Hyg. 2014;90:1124–32. 10.4269/ajtmh.13-0755. 24752686 10.4269/ajtmh.13-0755 PMC4047740

