# Correction: Double vision: 2D and 3D mosquito trajectories can be as valuable for behaviour analysis via machine learning

**DOI:** 10.1186/s13071-024-06410-6

**Published:** 2024-07-31

**Authors:** Yasser Mehmood Qureshi, Vitaly Voloshin, Catherine Elizabeth Towers, James Anthony Covington, David Peter Towers

**Affiliations:** 1 School of Engineering, University of Warwick, Coventry, CV4 7AL UK; 2 School of Biological and Behavioural Sciences, Queen Mary University of London, London, E1 4NS UK

**Correction: Parasites & Vectors (2024) 17:282** 10.1186/s13071-024-06356-9

Following publication of the original article [1], it came to the attention of the authors that the article had published with an out-of-date graphical abstract. Namely, the graphical abstract detailed results that the article had prior to peer review. The article has since been updated with the correct, up-to-date version of the graphical abstract, which includes revised values for ROC AUC, updated ROC curves, and an amended UMAP figure. The authors thank you for reading this erratum and apologize for any inconvenience caused.
