# Supplementary material for: Double vision: 2D and 3D mosquito trajectories can be as valuable for behaviour analysis via machine learning
Source: Parasit Vectors. 2024 Jul 1;17:282. doi: 10.1186/s13071-024-06356-9 (PMC11218242; doi:10.1186/s13071-024-06356-9)

Double Vision: 2D and 3D Mosquito Trajectories can be as Valuable for Behaviour Analysis via Machine Learning

Y.M. Qureshi, V. Voloshin, C.E. Towers, J.A. Covington, and D.P. Towers

**Supplementary Information**

**Fig. S1** SHAP summary plot for the best fold using the 3D dataset

**
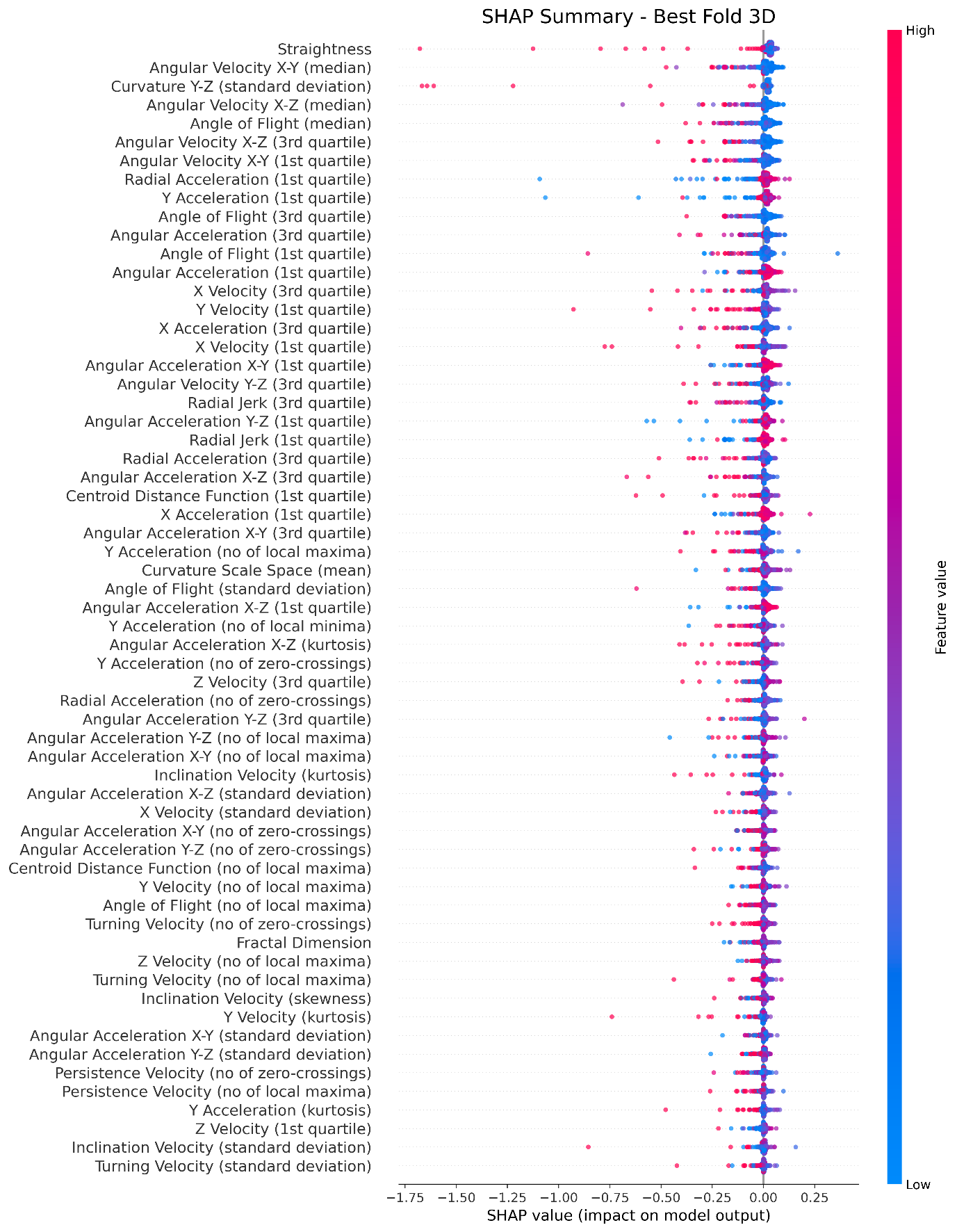
**

**Fig. S2** SHAP summary plot for the best fold using the 2D Telecentric dataset

**
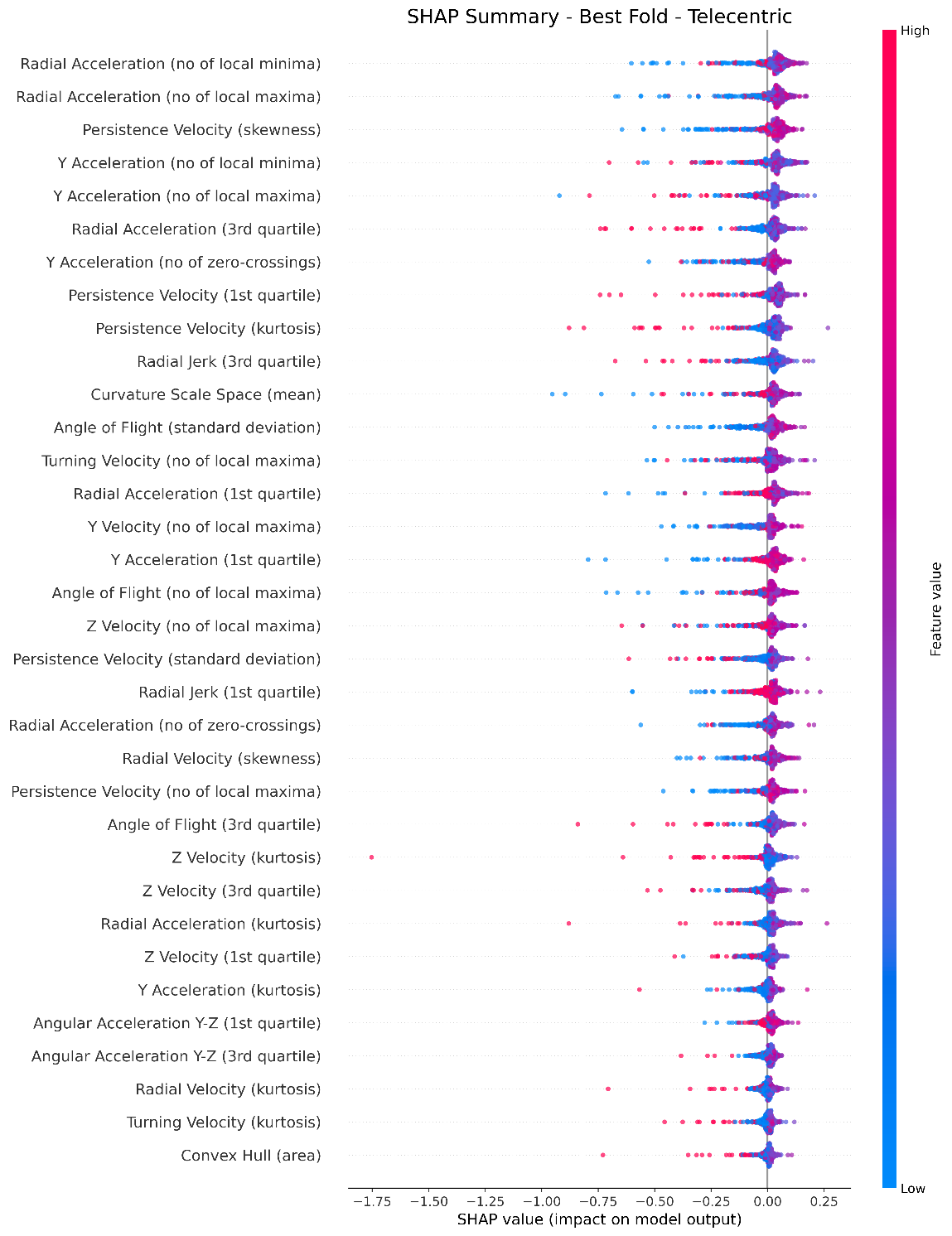
**

**Fig. S3** SHAP summary plot for the best fold using the 2D single camera at 2m dataset

**
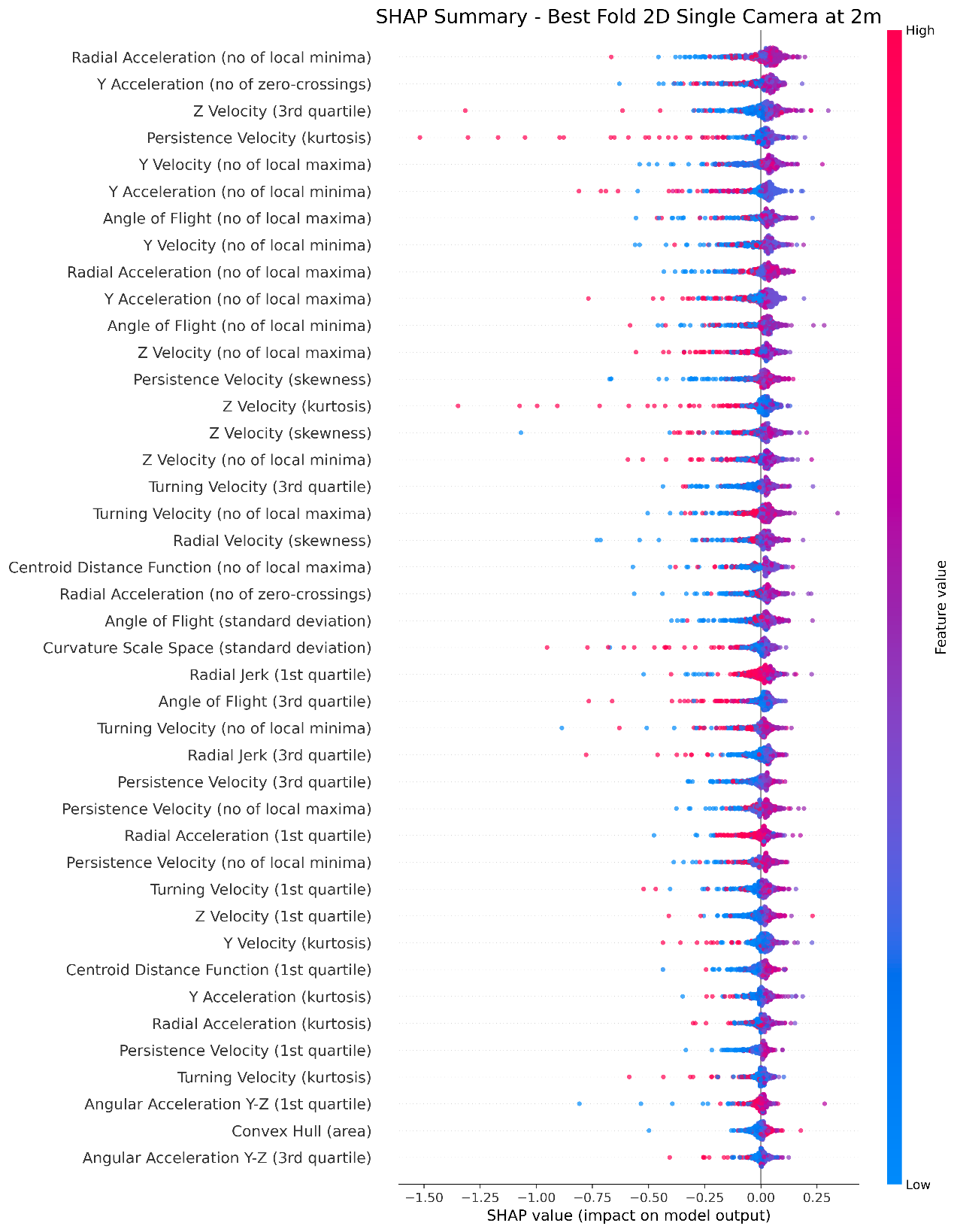
**

**Fig. S4** SHAP summary plot for the best fold using the 2D single camera at 15m dataset

**
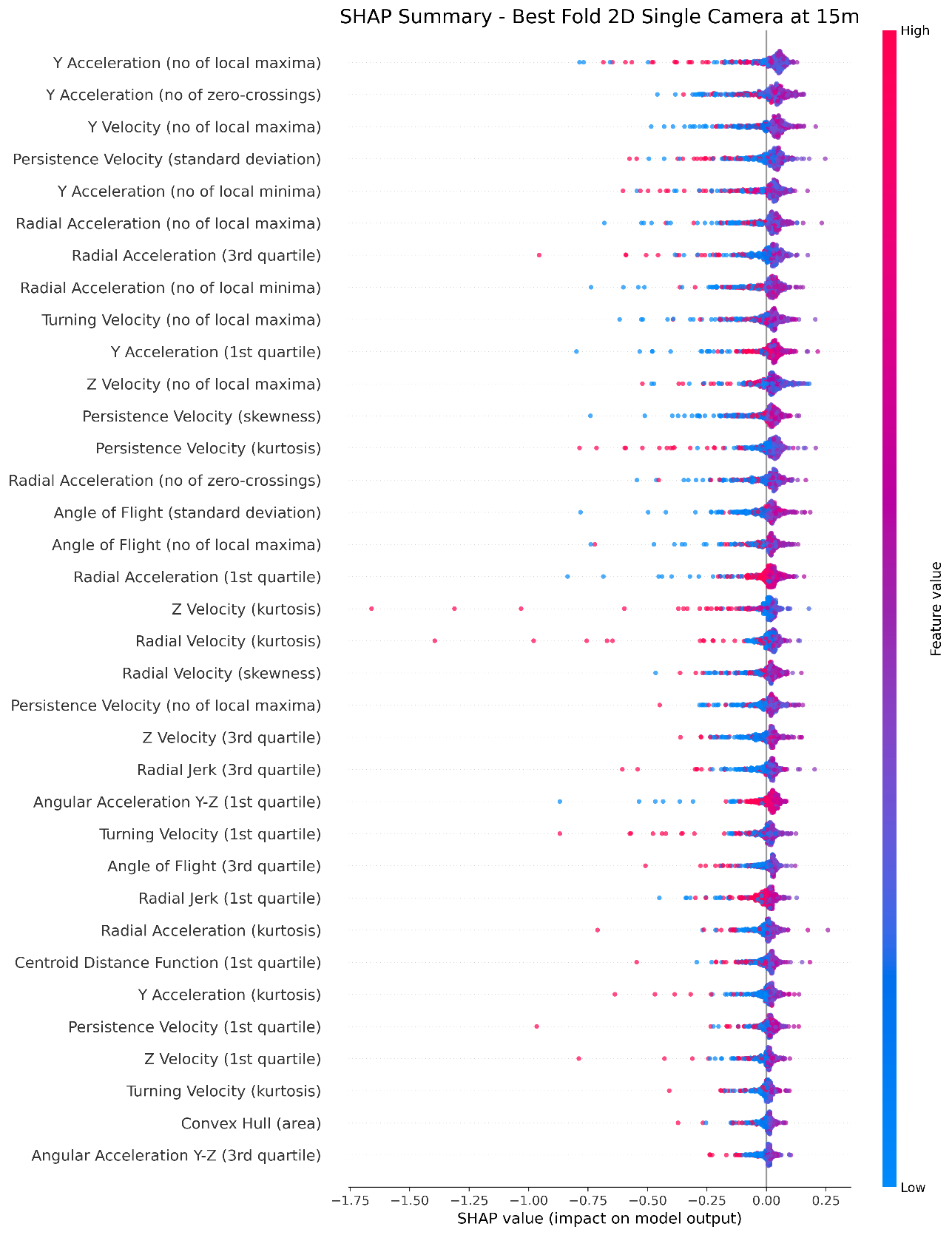
**

**Fig. S5** SHAP summary plot of the best fold using the 3D dataset only selecting the common features across all datasets sorted alphabetically

**
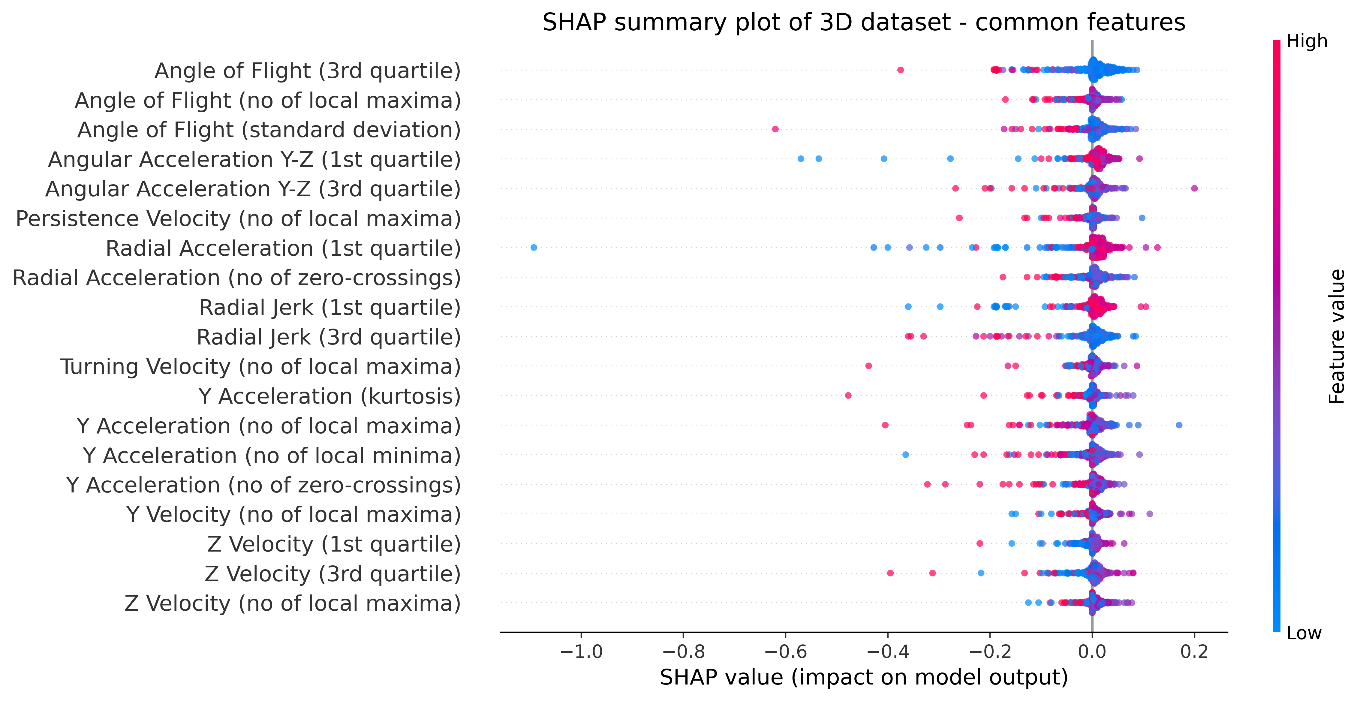
**

**Fig. S6** SHAP summary plot of the best fold using the 2D telecentric dataset only selecting the common features across all datasets sorted alphabetically

**
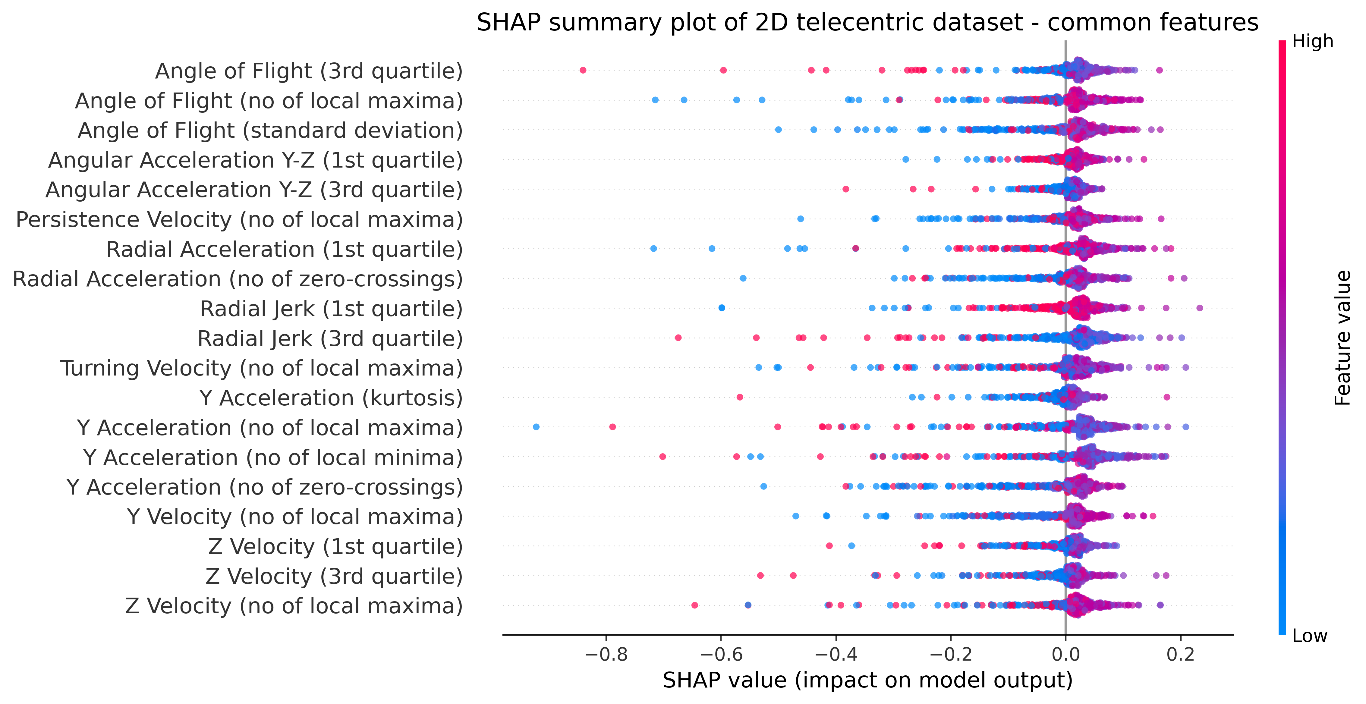
**

**Fig. S7** SHAP summary plot of the best fold using the 2D single camera at 2m dataset only selecting the common features across all datasets sorted alphabetically

**
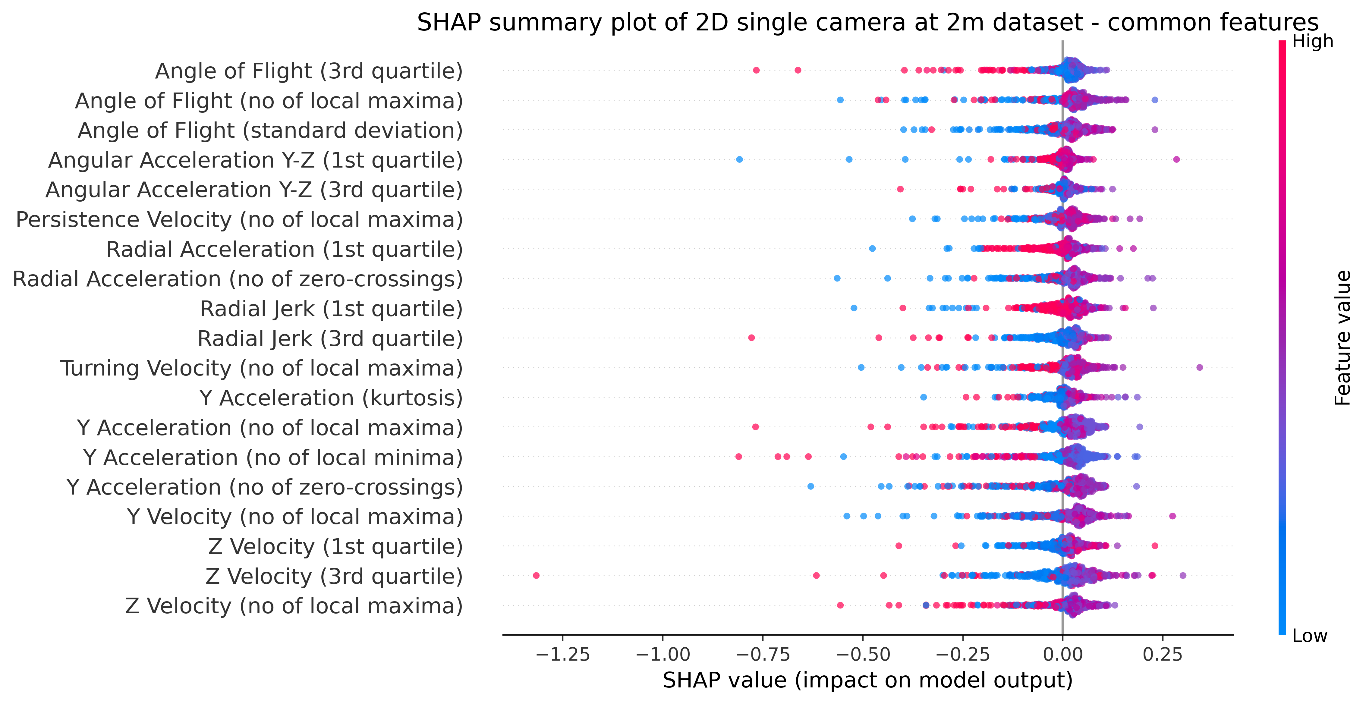
**

**Fig. S8** SHAP summary plot of the best fold using the 2D single camera at 15 m dataset only selecting the common features across all datasets sorted alphabetically

**
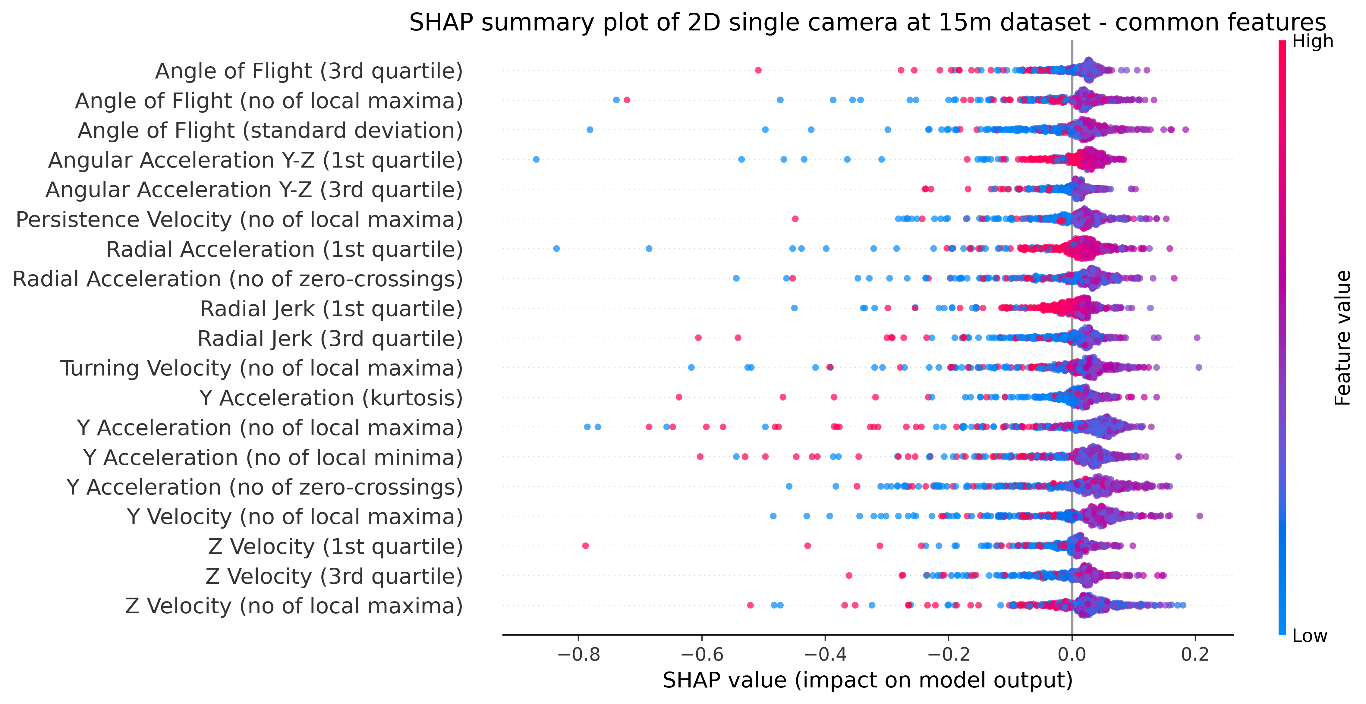
**

**Table. S9** Features extracted and their corresponding statistical features from 2D data

| **Feature Names** | **Statistical Properties** |
| --- | --- |
| Angle of Flight | - 1^st^ quartile - 3^rd^ quartile - Kurtosis - Mean - Median - Number of local minima - Number of local maxima - Number of zero crossings - Skewness - Standard deviation |
| Angular Acceleration | - 1^st^ quartile - 3^rd^ quartile - Kurtosis - Mean - Median - Number of local minima - Number of local maxima - Number of zero crossings - Skewness - Standard deviation |
| Angular Velocity | - 1^st^ quartile - 3^rd^ quartile - Kurtosis - Mean - Median - Number of local minima - Number of local maxima - Number of zero crossings - Skewness - Standard deviation |
| Centroid Distance Function | - 1^st^ quartile - 3^rd^ quartile - Kurtosis - Mean - Median - Number of local minima - Number of local maxima - Number of zero crossings - Skewness - Standard deviation |
| Convex Hull | - Area - Perimeter |
| Curvature Scale Space | - Mean - Standard deviation |
| Fractal dimension |  |
| Persistence Velocity | - 1^st^ quartile - 3^rd^ quartile - Kurtosis - Mean - Median - Number of local minima - Number of local maxima - Number of zero crossings - Skewness - Standard deviation |
| Radial Acceleration | - 1^st^ quartile - 3^rd^ quartile - Kurtosis - Mean - Median - Number of local minima - Number of local maxima - Number of zero crossings - Skewness - Standard deviation |
| Radial Jerk | - 1^st^ quartile - 3^rd^ quartile - Kurtosis - Mean - Median - Number of local minima - Number of local maxima - Number of zero crossings - Skewness - Standard deviation |
| Radial Velocity | - 1^st^ quartile - 3^rd^ quartile - Kurtosis - Mean - Median - Number of local minima - Number of local maxima - Number of zero crossings - Skewness - Standard deviation |
| Straightness |  |
| Turning Velocity | - 1^st^ quartile - 3^rd^ quartile - Kurtosis - Mean - Median - Number of local minima - Number of local maxima - Number of zero crossings - Skewness - Standard deviation |
| Y Acceleration | - 1^st^ quartile - 3^rd^ quartile - Kurtosis - Mean - Median - Number of local minima - Number of local maxima - Number of zero crossings - Skewness - Standard deviation |
| Y Velocity | - 1^st^ quartile - 3^rd^ quartile - Kurtosis - Mean - Median - Number of local minima - Number of local maxima - Number of zero crossings - Skewness - Standard deviation |
| Z Acceleration | - 1^st^ quartile - 3^rd^ quartile - Kurtosis - Mean - Median - Number of local minima - Number of local maxima - Number of zero crossings - Skewness - Standard deviation |
| Z Velocity | - 1^st^ quartile - 3^rd^ quartile - Kurtosis - Mean - Median - Number of local minima - Number of local maxima - Number of zero crossings - Skewness - Standard deviation |

**Table. S10** Hyperparameter tuning parameter ranges

| **Hyperparameter** | **Range** | **Step size** |
| --- | --- | --- |
| Kernel | Linear; Radial basis function; Polynomial; sigmoid | - |
| Window size | $0.4-3 s$ | $0.08 s$ |
| Overlap length | $0.2-3 s$ | $0.08 s$ |

**Table. S11** Selected hyperparameters for each dataset after tuning for the Y-Z plane, approximately parallel to the camera detectors.

| **Camera System** | **Distance** | **Kernel** | **Window size (s)** | **Overlap length (s)** |
| --- | --- | --- | --- | --- |
| single camera | 2000 | rbf | 1.12 | 0.2 |
| single camera | 2500 | rbf | 1.12 | 0.2 |
| single camera | 3000 | rbf | 1.04 | 0.2 |
| single camera | 3500 | rbf | 1.36 | 0.2 |
| single camera | 4000 | rbf | 1.04 | 0.2 |
| single camera | 4500 | rbf | 0.88 | 0.36 |
| single camera | 5000 | rbf | 1.04 | 0.2 |
| single camera | 5500 | rbf | 0.8 | 0.28 |
| single camera | 6000 | rbf | 1.12 | 0.2 |
| single camera | 6500 | rbf | 1.12 | 0.2 |
| single camera | 7000 | rbf | 1.28 | 0.36 |
| single camera | 7500 | rbf | 1.12 | 0.2 |
| single camera | 8000 | rbf | 1.04 | 0.2 |
| single camera | 8500 | rbf | 1.2 | 0.28 |
| single camera | 9000 | rbf | 1.44 | 0.28 |
| single camera | 9500 | rbf | 1.44 | 0.28 |
| single camera | 10000 | rbf | 1.44 | 0.28 |
| single camera | 10500 | rbf | 1.44 | 0.28 |
| single camera | 11000 | rbf | 1.44 | 0.28 |
| single camera | 11500 | rbf | 1.44 | 0.28 |
| single camera | 12000 | rbf | 1.44 | 0.28 |
| single camera | 12500 | rbf | 1.44 | 0.28 |
| single camera | 13000 | rbf | 1.44 | 0.28 |
| single camera | 13500 | rbf | 1.44 | 0.28 |
| single camera | 14000 | rbf | 1.44 | 0.28 |
| single camera | 14500 | rbf | 1.44 | 0.28 |
| single camera | 15000 | rbf | 1.44 | 0.28 |
| telecentric | - | rbf | 1.44 | 0.28 |
| 3D | - | rbf | 1.92 | 0.84 |

**Fig. S12** Performance as distance increases for the X-Y plane (top view). (a) Displays balanced accuracy and (b) displays ROC AUC score.


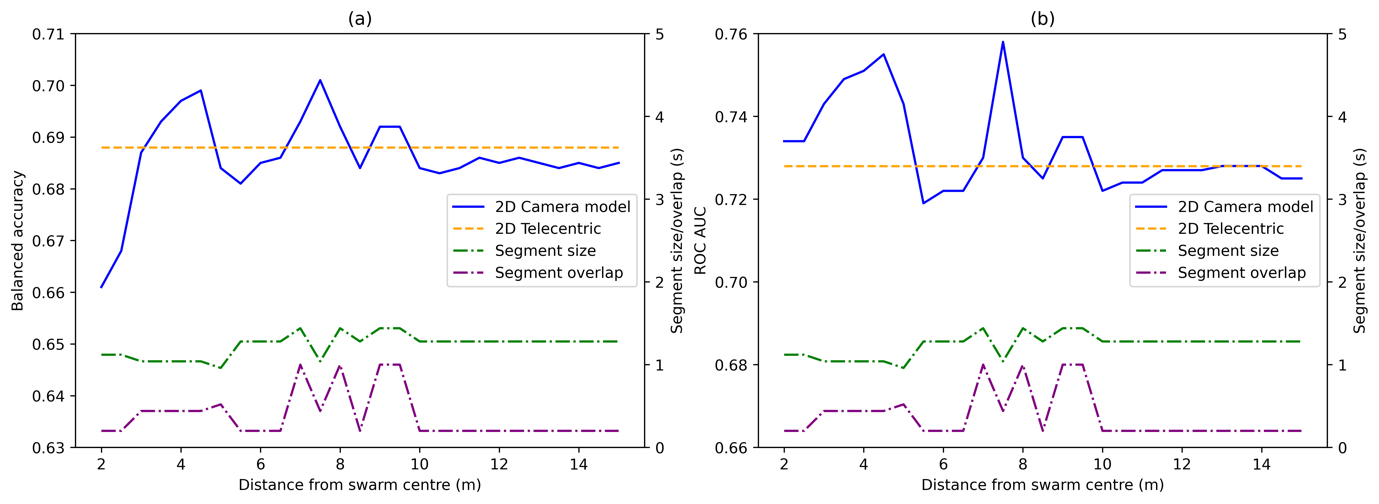


**Fig. S13** Performance as distance increases for the X-Z plane (other side view). (a) Displays balanced accuracy and (b) displays ROC AUC score.


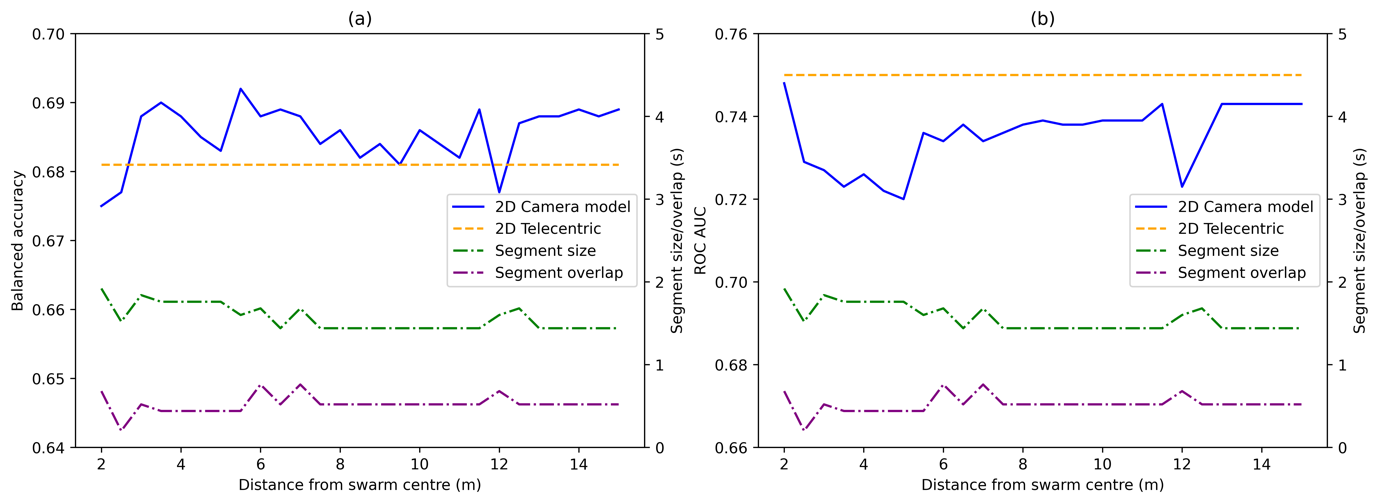

Supplement: Supplementary file 1 — Additional file 1: Figure S1. SHAP summary plot for the best fold using the 3D dataset. Figure S2. SHAP summary plot for the best fold using the 3D dataset. Figure S3. SHAP summary plot for the best fold using the 3D dataset. Figure S4. SHAP summary plot for the best fold using the 2D single camera at 15 m dataset. Figure S5. SHAP summary plot of the best fold using the 3D dataset only selecting the common features across all datasets sorted alphabetically. Figure S6. SHAP summary plot of the best fold using the 2D telecentric dataset only selecting the common features across all datasets sorted alphabetically. Figure S7. SHAP summary plot of the best fold using the 2D single camera at 2 m dataset only selecting the common features across all datasets sorted alphabetically. Figure S8. SHAP summary plot of the best fold using the 2D single camera at 15 m dataset only selecting the common features across all datasets sorted alphabetically. Table S9. Features extracted and their corresponding statistical features from 2D data. Table S10. Hyperparameter tuning parameter ranges. Table S11. Selected hyperparameters for each dataset after tuning. Figure S12. Performance as distance increases for the X–Y plane (top view). (a) Displays balanced accuracy and (b) displays ROC AUC score. Figure S13. Performance as distance increases for the X–Z plane (other side view). (a) Displays balanced accuracy and (b) displays ROC AUC score. [file 13071_2024_6356_MOESM1_ESM.docx]
